# *In vivo* imaging of clock gene expression in multiple tissues of freely moving mice

**DOI:** 10.1038/ncomms11705

**Published:** 2016-06-10

**Authors:** Toshiyuki Hamada, Kenneth Sutherland, Masayori Ishikawa, Naoki Miyamoto, Sato Honma, Hiroki Shirato, Ken-ichi Honma

**Affiliations:** 1 Department of Applied Molecular-Imaging Physics, Hokkaido University Graduate School of Medicine, Sapporo 060-8638, Japan; 2 Department of Medical Physics and Engineering, Hokkaido University Graduate School of Medicine, Sapporo 060-8638, Japan; 3 Department of Biomedical Science and Engineering, Hokkaido University Graduate School of Health Science, Sapporo 062-0812, Japan; 4 Global Station for Quantum Medical Science and Engineering, Global Institution for Collaborative Research and Education (GI-CoRE), Hokkaido University, Sapporo 060-8638, Japan; 5 Department of Chronomedicine, Hokkaido University Graduate School of Medicine, Sapporo 060-8638, Japan; 6 Department of Radiation Medicine, Hokkaido University Graduate School of Medicine, Sapporo 060-8638, Japan

## Abstract

Clock genes are expressed throughout the body, although how they oscillate in unrestrained animals is not known. Here, we show an *in vivo* imaging technique that enables long-term simultaneous imaging of multiple tissues. We use dual-focal 3D tracking and signal-intensity calibration to follow gene expression in a target area. We measure circadian rhythms of clock genes in the olfactory bulb, right and left ears and cortices, and the skin. In addition, the kinetic relationship between gene expression and physiological responses to experimental cues is monitored. Under stable conditions gene expression is in phase in all tissues. In response to a long-duration light pulse, the olfactory bulb shifts faster than other tissues. In *Cry1*^*−/−*^ *Cry2*^*−/−*^ arrhythmic mice circadian oscillation is absent in all tissues. Thus, our system successfully tracks circadian rhythms in clock genes in multiple tissues in unrestrained mice.

## Abstract (web summary)

The circadian rhythms of peripheral clocks are difficult to study. Here the authors demonstrate a technique to image clock gene expression simultaneously in various tissues of freely moving mice, and use it to show that a long duration light pulse resets the rhythms in the olfactory bulb faster than other tissues.

## Graphical abstract

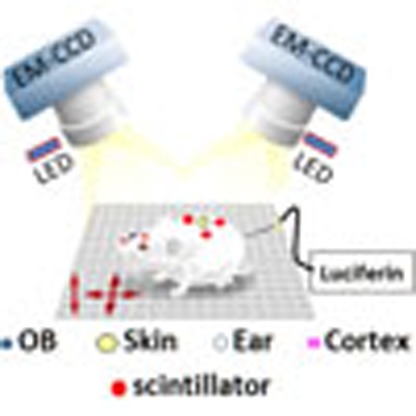

Circadian rhythms in physiology and behaviour in mammals are regulated by a master clock located in the suprachiasmatic nucleus (SCN). Clock genes have critical roles in expressing cellular circadian rhythms, primarily based on a cell autonomous molecular feedback loop^1^. Clock gene expression is not limited to the SCN, with expression observed in a variety of tissues^1^,^2^,^3^,^4^,^5^. However, the expression pattern of clock genes outside the SCN is not well understood, partly owing to a lack of techniques for simultaneous monitoring of circadian clock gene rhythms in specific tissues, and to detect output functions in the absence of physical restraints.

The bioluminescent reporter enzyme firefly *luciferase* (*luc*) and its substrate D-luciferin (luciferin) have been used to generate optical imaging signals with high sensitivity in living animals^6^, and have been adapted successfully to whole-body imaging of clock genes in anaesthetized mice using a charge-coupled device (CCD) camera^7^,^8^. However, anaesthesia is reported to alter the clock gene expression^9^,^10^,^11^,^12^,^13^,^14^,^15^. Saini *et al*.^16^ measured the rhythmic *bmal1* expression only in the liver of a freely moving mouse with a CCD camera located outside the body.

Monitoring of clock gene expression *in vivo* has also been attempted using a bioluminescent reporter, either with an optical fibre in the SCN^17^, or using a photon detector located outside the body. In the latter case, bioluminescence emitted from tissue-specific reporters was collected efficiently using a conical wall that channels photons toward a photomultiplier tube (PMT), and the emitting tissue was identified using a tissue-specific reporter^16^. Although promising, these approaches have limitations. For example, monitoring clock gene expression using an implanted optical fibre only enables focusing on the target area in a limited space, and insertion of an optical fibre may damage the area surrounding the target^18^. In the case of bioluminescent detection using a PMT external to the body, tissue-specific bioluminescence may need to be quantified if the target is relatively large and is located in deep areas inside the body. In addition, three-dimensional (3D)-calibration of signal strength is necessary.

Here, we show a method to quantitatively monitor clock gene expression simultaneously in multiple regions of freely moving mice over long durations. The challenge was to calibrate the bioluminescence intensity in moving targets. To overcome the technical challenge of intensity changes with time-of-day and distance from the recording apparatus, we developed a dual-focal 3D tracing (DuFT) technology and a signal-intensity calibration technique (SICT) and combined these two systems into a software application for analysing gene expression, which we call ‘Mouse Tracker'.

These techniques enable us to monitor the circadian rhythm of clock gene expression concurrently in multiple tissues with unrestrained mice. The results indicate that the olfactory bulb (OB) shifts faster than other tissues (right and left ears and cortices, and skin) in response to a long-duration light pulse.

## Results

**Figure 1:**
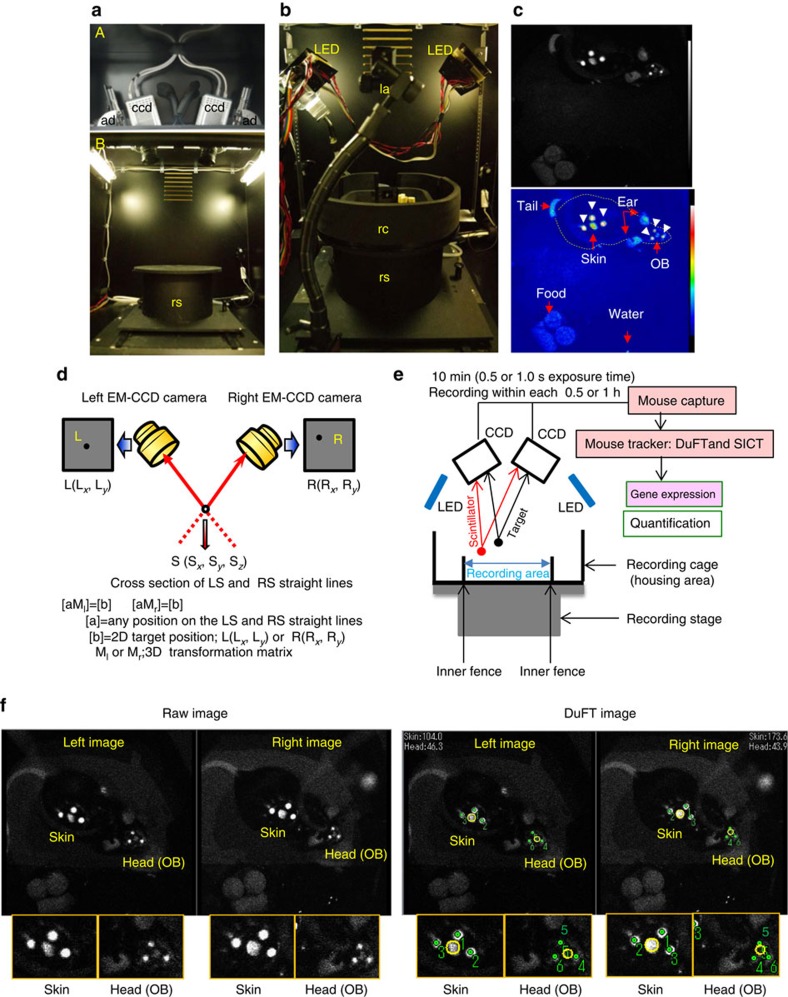
*In vivo* imaging system. (**a**) Inside the EM-CCD camera box (A) and imaging box (B). The upper photo shows two high-sensitive EM-CCD cameras (ccd) with an angle adjuster (ad). The lower photo shows two fluorescent lights and the recording stage (rs) in the ventilated imaging box. (**b**) DuFT recording system. Recording cage (rc) on the recording stage, two LED lights and luciferin application system (la) are shown. (**c**) *Per1-luc* mouse with scintillators attached to the head and skin exposed to the LED light in freely moving condition. The fluorescence signal from the scintillators and bioluminescent signals from the target area are acquired by EM-CCD cameras in the raw data (upper) and pseudo-colour data (lower). The arrowhead shows three scintillators around the skin target and those around the OB targets. The dotted line shows the shape of the mouse body. Luciferin (40 mg ml^−1^) was supplied by an iPRECIO pump (15 μl h^−1^). Images were captured at 0.5 s per frame. (**d**) Determination of the 3D coordinates of a scintillator {S (S_*x*_, S_*y*_, S_*z*_)} in the recording cage. The 3D transformation matrix from the 2D coordinates (*x*-axis limitation; 0<*x*<512, *y*-axis limitation; 0<*y*<512) on a pair of images {left; L (L*x*, L*y*) and right; R (R*x*, R*y*)} was used to make a straight line passing through points L and S or R and S. The 3D coordinates of the scintillator {S (S_*x*_, S_*y*_, S_*z*_)} are calculated by intersecting these two lines. (**e**) *In vivo* imaging system overview. Data are saved in the computer at each 0.5 or 1 h. Saved data are analysed by ‘Mouse Tracker' consisting of pattern match program, tracking program and 3D calibration program. Quantification analysis yields the gene expression levels. (**f**) Representative image of *Per1* expression of freely moving *Per1-luc* transgenic mouse by two EM-CCD cameras. DuFT image is made by analysing the raw image with ‘Mouse Tracker'. Three scintillators on the head and skin are identified by DuFT. Green spots in the image indicate the scintillator positions determined by the software, numbered by descending pattern-matching score.

The *in vivo* imaging system used in this study consists of two novel techniques, DuFT and SICT (Fig. 1a,b and Supplementary Fig. 1a–g). Bioluminescent signals representing *Per1* expression were produced following intraperitoneal administration of luciferin through an application system (Fig. 1b and Supplementary Fig. 1d,e) into freely moving *Per1*-*luc* mice (Fig. 1c). The following sections describe the principles and detailed methods of each technology.

### DuFT for detecting the 3D positions of scintillators

To serve as position markers, scintillators attached to the skin of the back (hereafter referred as skin) and the head were used to detect the regions of interest (ROIs), namely OB, ears and cerebral cortices (hereafter referred as cortex; Fig. 1c). To detect the 3D positions of a scintillator in freely moving animals in the recording cage (Supplementary Fig. 1f), we developed tracking software based on stereo photography^19^,^20^,^21^ and pattern-matching^22^ principles. Two cameras captured a pair of images (left; L (L*x*, L*y*) and right R (R*x*, R*y*)). From the 2D coordinates of the scintillators in each camera's image, two straight lines passing through a scintillator were calculated using transformation matrices (M_l_ and M_r_; Fig. 1d) (see the ‘Methods' section for details). The 3D coordinates of the scintillator (S (S_*x*_, S_*y*_, S_*z*_)) were determined by calculating the intersection of these two lines (Fig. 1d). In situations when the signal of the scintillator is lost due to the angle of the two EM-CCD cameras, the signal can be reacquired automatically when the mouse moves into a more appropriate position. Intensity of the LED-light-induced fluorescent signals (Supplementary Fig. 2a–c) from the scintillators was set (Supplementary Fig. 1g) to be roughly equivalent to the bioluminescent signals from the skin of the freely moving mice (Fig. 1c).

### Signal-intensity calibration technique

**Figure 2:**
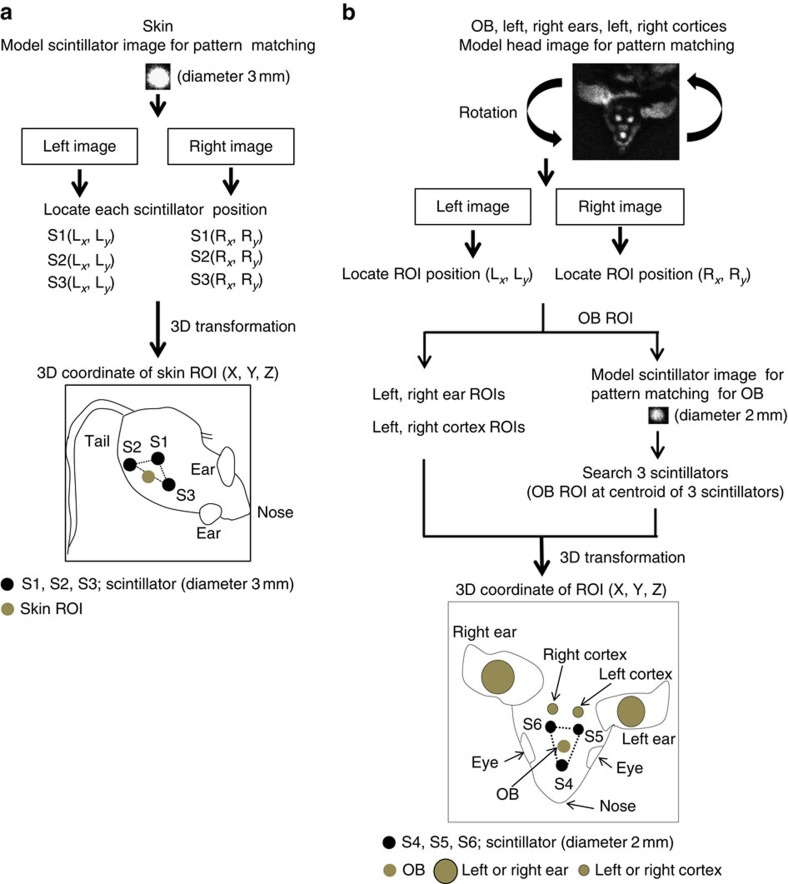
Determination of ROI positions in six areas of freely moving mice. First, the frames with OB, skin, ear, cortex and three scintillators on each image are selected by ‘Mouse Tracker'. (**a**) For skin, three scintillator ROIs are identified using a model scintillator image. The skin ROI was assumed to be at the centre of the baseline of the triangle formed by the three scintillators. (**b**) For OB, ear and cortex ROIs, a model head image is used as a search model for pattern matching in all frames of the video with any angle of rotated versions of the model. Ear and cortex ROIs are defined relative to the top-left corner of the model head image. The OB ROI was assumed to be at the centroid of the triangle formed by the three scintillators. Next, 3D coordinates of ROI in each area are computed by transformation matrix (see the ‘Methods' section for details). 3D coordinates of ROIs are illustrated in the skin and head of the mouse.

We developed a number of algorithms to locate the scintillators using a model image for pattern matching to identify the associated ROIs. For the skin, scintillators were attached on a patch of shaved skin. The ROI was assumed to be at the centre of the baseline of the triangle formed by the three scintillators. The OB ROI was assumed to be at the centroid of the triangle formed by the three scintillators (Fig. 1f). For the head, an image of one of the three scintillators placed on the head was used as a model for pattern matching. To identify left and right ears/cortices (Supplementary Fig. 3), we further developed a method using a model head image for pattern matching (Fig. 2a,b). Adding the model head image for pattern matching resulted in decreased mismatching of the scintillator image. Selection of the model head image for pattern matching (model image in Supplementary Fig. 4 is for OB and model image in Fig. 2b is for OB, left ear, right ear, left cortex, right cortex) is based on the number of target areas to be measured.

The intensity of the bioluminescent signal varies with the position of the animal within the recording cage due to variations in distance from the camera. To calibrate the bioluminescent signal obtained from individual ROIs, we calibrated signal intensity using the 3D coordinates of the target area on the mouse by referring to a calibration table (Supplementary Fig. 5a–f; see the ‘Methods' section for details) which specifies the ratio of the calibration value at each point in the imaging space.

The bioluminescence from the target area was calculated from the mean ROI pixel intensity (mean pixel value; shown with a yellow circle in Fig. 1f) for each image from the EM-CCD cameras. In this way, *Per1* expression in ROIs was automatically marked with circles (identification), traced (Supplementary Movie 1) and quantified (Supplementary Fig. 6).

The spatial resolution was estimated from the cage positioning and the pattern-matching errors. The maximum error in cage positioning was 0.21 mm which was calculated from 1,815 measurements of 605 different points (each 3 times) in the cylindrical matrix (r, 5 cm; height, 4 cm; Supplementary Fig. 5). We estimated the cage positioning error to be 0.63 mm which was 3 times of the largest standard deviation (s.d.) of the mean. The pattern-matching error was calculated to be 1.50 mm (3 s.d.), when matching of >30% was accepted as valid. Thus, the spatial resolution with a confidence range of 99.9% is 1.63 mm.

### *Per1* and *Bmal1* expression in OB and skin of transgenic mice

**Figure 3:**
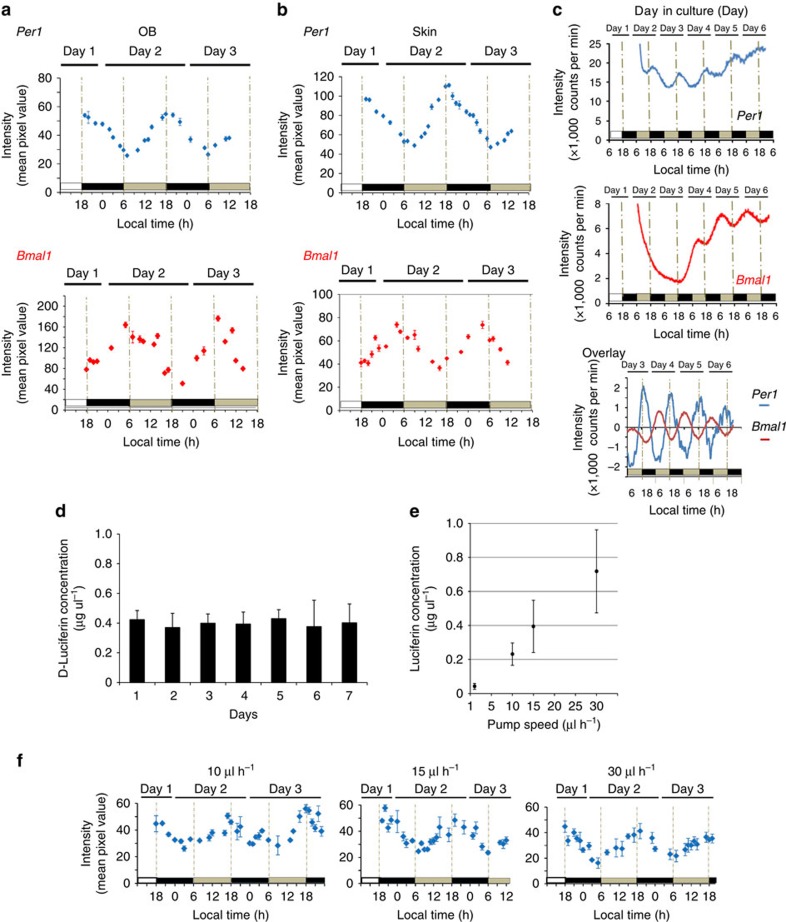
Circadian *Per1* and *Bmal1* expression rhythm of the transgenic mice. *In vivo* imaging of *Per1* (**a**) and *Bmal1* (**b**) expression from OB and skin in freely moving mice. Luciferin was supplied by an iPRECIO pump (15 μl h^−1^ for *Per1*-*luc* and 30 μl h^−1^ for *Bmal1*-enhanced beetle *luciferase* (*ELuc*)). Images were captured for 10 min, at 0.5 s per frame for *Per-luc* and 1.0 s per frame for *Bmal1*-*ELuc*, at 1-h intervals. (**c**) *Ex vivo* imaging of *Per1* and *Bmal1* expression from the OB. *Per1-luc* and *Bmal1-ELuc* activities in the horizontal whole bilateral OB slices recorded for 1 min at every 10 min intervals for 6 days. The abscissa indicates days in culture. Day 0 is the day of slice preparation. The baseline-corrected bioluminescence was computed by subtracting the 24-h moving average from the original data. (**d**) Luciferin concentration (mean±s.d.) in the plasma for 7 days. Luciferin concentration in plasma was stable for 7 days when luciferin (40 mg ml^*−*1^) was supplied at a perfusion rate of 15 μl h^−1^ using an iPRECIO pump. (**e**) The relationship between luciferin concentration (mean±s.d.) and perfusion rate. (**f**) Rhythmic *Per1* expression of the transgenic mice *in vivo* at various perfusion rates (40 mg ml^*−*1^). *Per1-luc* mice implanted with an osmotic pump (1 μl h^−1^) into the peritoneal cavity exhibited low plasma luciferin concentration of 0.04±0.02 μg μl^−1^, and did not show detectable bioluminescent signals.

*Per1* and *Bmal1* expression rhythms *in vivo*, which was compared with *ex vivo* expression in the OB and skin of freely moving mice under constant darkness (DD) are shown in Fig. 3a,b. In the OB and skin, circadian rhythms in *Per1* gene expression were robust, exhibiting peak expression in the subjective evening as previously reported^23^,^24^. In contrast, *Bmal1* showed circadian gene expression that was antiphasic to that of *Per1*. The OB results were consistent with *ex vivo* experiments (Fig. 3c) in which *Per1* and *Bmal1* expression showed a similar antiphasic relationship in horizontal brain slices of the whole OB (0.3 mm slice thickness). Circadian peak and trough of *Per1-luc* expression rhythm on the third culture day were observed at 18:12±0:36 and 5:12±0:48 local time (mean±s.d., *n*=3, period=23.4±0.3 h), respectively. Whereas, the peak and trough of *Bmal1–ELuc* expression rhythm on the fourth culture day were observed at 5:00±1:30 and 15:15±0:54 (mean±s.d., *n*=3, period=22.6±0.3 h). These results were consistent with our previous *in vivo* study of mRNA expression^23^.

Supplementary Movies 2 and 3 show *Per1* gene expression at 19:00 hours and 07:00 hours, respectively on the first day in DD. The recorded target areas were OB and skin. At 19:00 hours the mouse was eating food and moving around in the cage. Representative images at each time point are shown in Supplementary Fig. 7a. The 3D plots tracking the OB show behaviour in the cage at each time point (Supplementary Fig. 7b). At 07:00 hours in the morning, the mouse was inactive and remained in one position (Supplementary Fig. 7b). Calibrated bioluminescent intensity of *Per1-luc* expression in the OB and skin of freely moving mice in the recording cage were confirmed by extracting the data in which the mouse was stationary during a 10-s period (changes in spatial position of ≤1 mm; Supplementary Fig. 8).

### Continuous luciferin administration

To quantify the bioluminescent signal at the whole-body level for an extended period of time, the luciferin concentration in blood should be stable over the recording period. In the present study, the plasma luciferin concentration was maintained at a stable level for 7 days (Fig. 3d). By maintaining the plasma luciferin concentration over 0.2 μg μl^−1^, we successfully detected rhythmic bioluminescent emission in freely moving mice (Fig. 3e,f and Supplementary Fig. 9a–d). Using an osmotic or iPRECIO pump, *Per1-luc* mice were administered luciferin at 1, 10, 15 or 30 μl hr^−1^, and the plasma luciferin concentration in each mouse was 0.04±0.02, 0.23±0.07, 0.39±0.15 and 0.72±0.24 μg μl^−1^, respectively (Fig. 3e).

Plasma luciferin concentrations increased depending on the speed of dosage administration, and we were able to obtain images of *Per1* (0.5 s EM-CCD camera exposure time) and monitor circadian *Per1* expression in freely moving mice at plasma luciferin levels above 0.2 μg μl^−1^ (Fig. 3f). Under these conditions, plasma luciferin concentrations were stable over a 24-h period. Therefore, we chose conditions where plasma luciferin concentrations exceeded 0.2 μg μl^−1^.

### *Per1-luc* expression in freely moving wild-type mice

**Figure 4:**
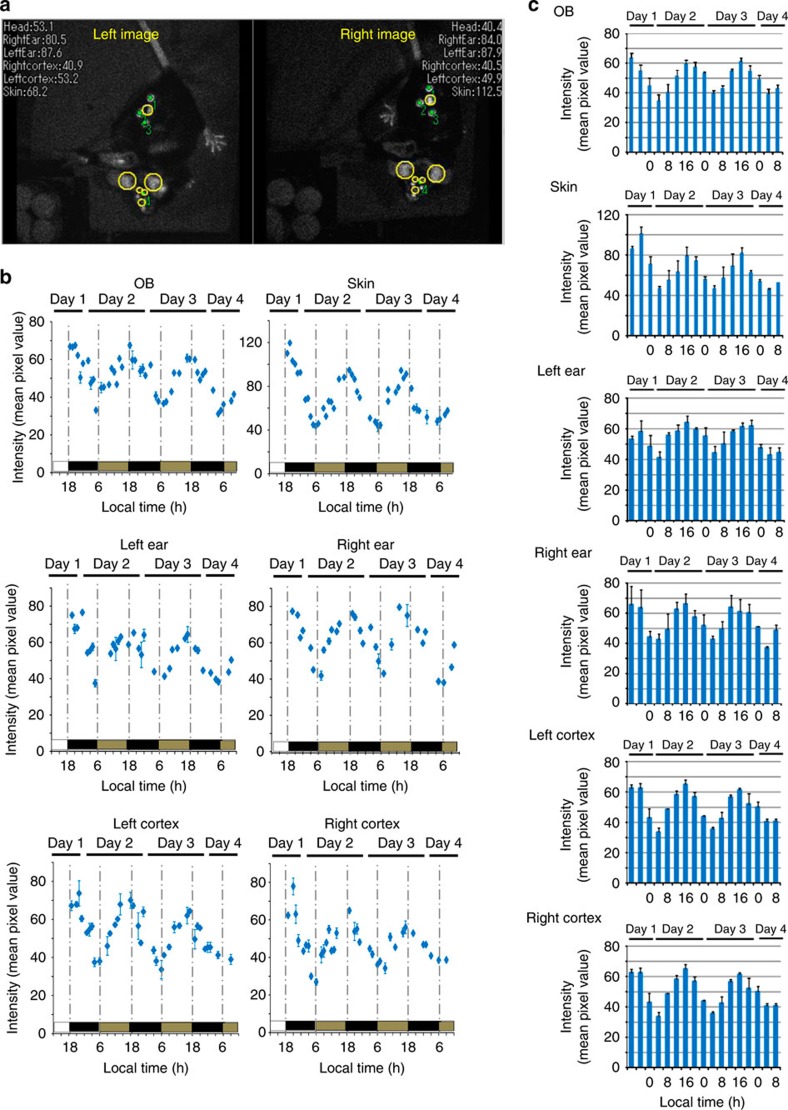
*In vivo* imaging of *Per1* expression in six areas of *Per1-luc* transgenic mice. Target areas (OB, skin, left ear, right ear, left cortex and right cortex) of *Per1-luc* (**a**) are automatically identified and traced. Yellow circles indicate identified areas by both EM-CCD cameras. Tracking marks and raw intensity within yellow circle areas are displayed in real time in the videos (30% correlation). To quantify the gene expression in the ROI, mismatched frames were discarded during post-processing (see the ‘Methods' section for details). Green spots in the image indicate the scintillator positions determined and numbered by the software in descending order of pattern matching score. (**b**) Representative *Per1* expression rhythm in 6 areas of *Per1-luc* mice. Luciferin was supplied by an iPRECIO pump (30 μl h^−1^ for animals). (**c**) *Per1* expression in six areas of *Per1-luc* transgenic mice. The mean bioluminescence (4 h mean±s.e.m.) in six areas (*n*=3̃4 animals). *Per1* expression in six areas exhibited robust circadian rhythm.

*Per1-luc* expression in the OB, skin, left ear, right ear, left cortex and right cortex was measured continuously for several days in freely moving mice under DD^23^,^24^,^25^ (Fig. 4a,b and Supplementary Movie 4). Circadian rhythms in *Per1* gene expression were detected in all ROIs examined with a peak during the early night and a trough during the early morning (Fig. 4c). In all *Per1-luc* mice examined, the six ROIs showed similar circadian phases in *Per1* expression. Circadian rhythmicity of quantified *Per1* expression analysed by the Cosinor program^26^ (see the ‘Methods' section for details) is shown in Supplementary Tables 1 and 2.

### *Per1-luc* expression in freely moving *Cry1* ^*−/−*^ *Cry2* ^*−/−*^ mice

**Figure 5:**
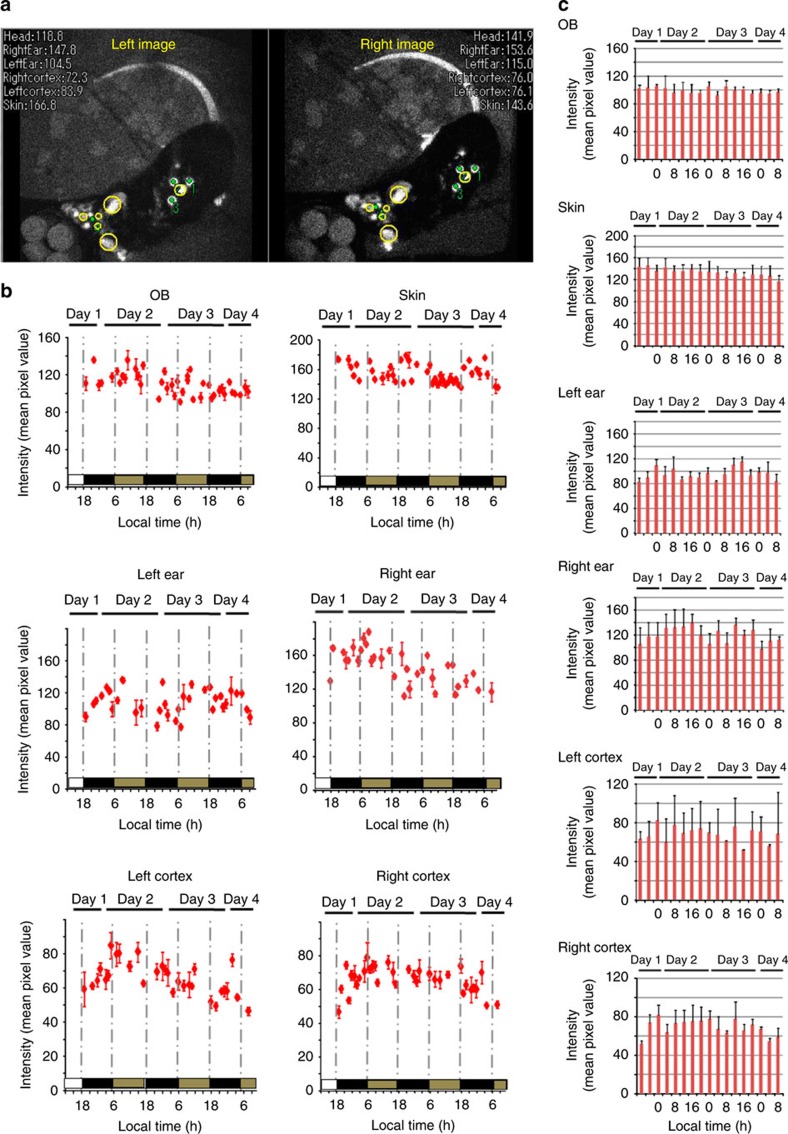
*In vivo* imaging of *Per1* expression in six areas of *Cry1*^*−*/*−*^/*Cry2*^*−*/*−*^-*Per1-luc* mice. (**a**) *Per1* expression in the six different areas of the *Cry1*^*−*/*−*^/*Cry2*^*−*/*−*^-*Per1-luc* mice. Target areas (OB, skin, left ear, right ear, left cortex and right cortex) of *Cry1*^*−*/*−*^/*Cry2*^*−*/*−*^-*Per1-luc* mice were automatically identified and traced. Yellow circles indicate identified areas by both EM-CCD cameras. Tracking marks and raw intensity within yellow circle areas are displayed in real time in the videos (30% correlation). To quantify the gene expression in the ROI, mismatched frames were discarded during post-processing.(see the ‘Methods' section for details). Green spots in the image indicate the scintillator positions determined and numbered by the software in descending order of pattern matching score. Green spots in the image indicate the scintillator positions determined and numbered by the software in descending order of pattern matching score. (**b**) Representative *Per1* expression rhythm in six areas of *Cry1*^*−*/*−*^/*Cry2*^*−*/*−*^-*Per1-luc* mice. Luciferin (40 mg ml^−1^) was applied into the intraperitoneal cavity by an iPRECIO pump (30 μl hr^−1^ for animals). (**c**) *Per1* expression in six areas of *Cry1*^*−*/*−*^/*Cry2*^*−*/*−*^-*Per1-luc* transgenic mice. The mean bioluminescence (4 hr mean±s.e.m.) in six areas (*n*=3̃4 animals). *Per1* expressions in six areas show no circadian rhythm.

We examined *Per1-luc* in *Cryptochrome 1* (*Cry1*) and *Cryptochrome 2* (*Cry2*) double-knockout (*Cry1*^*−*/*−*^/*Cry2*^*−*/*−*^ -*Per1-luc*) mice. *Cry1* and *Cry2* are components of a circadian feedback loop that generates circadian oscillation^27^. Adult *Cry1*^*−*/*−*^/*Cry2*^*−*/*−*^-*Per1-luc* mice are known to be aperiodic in behaviour and clock gene expression in the SCN under DD. As demonstrated in Fig. 5a–c and Supplementary Movie 5, *Cry1*^*−*/*−*^/*Cry2*^*−*/*−*^-*Per1-luc* mice did not exhibit a significant circadian rhythm in *Per1* expression with higher expression levels observed throughout the day, compared with the wild-type mice, in all ROIs examined (Supplementary Table 3). This result excludes the possibility that the observed 24-h rhythm is an artefact.

### Effects of a light pulse on circadian *Per1-luc* rhythms

**Figure 6:**
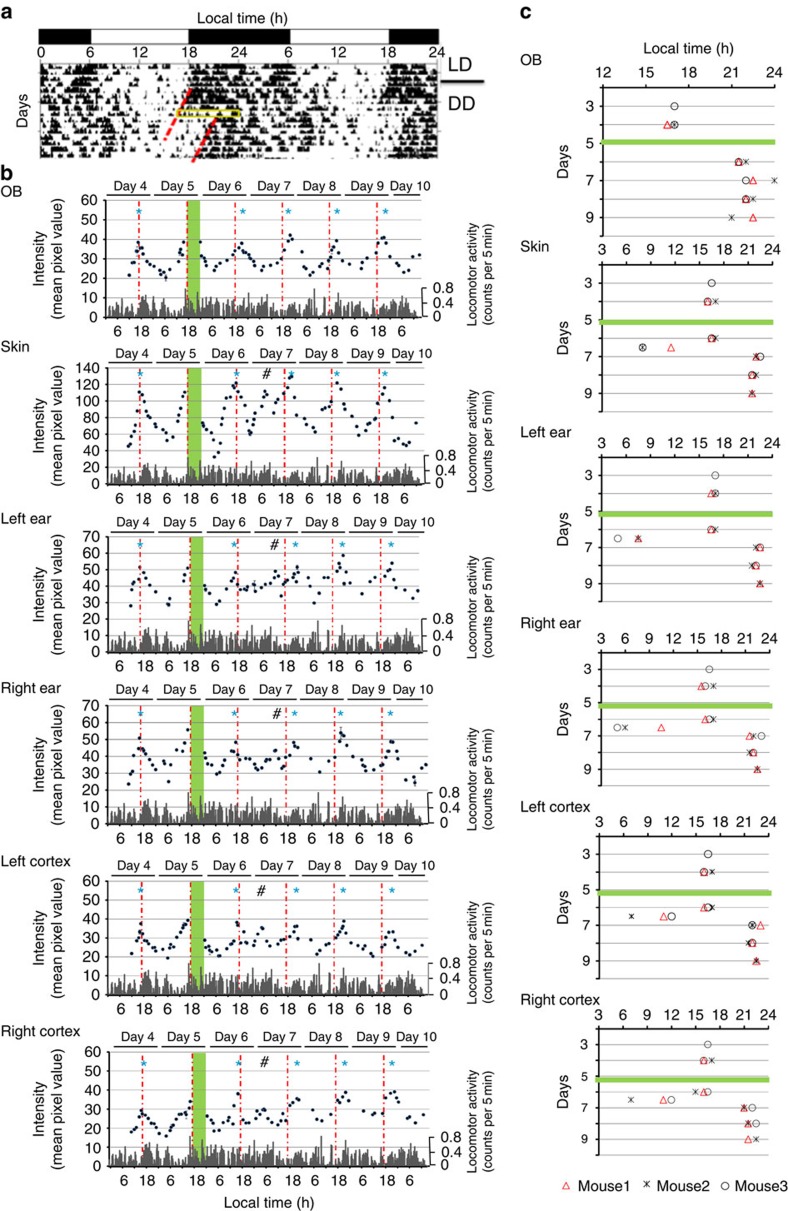
Different rate of photic phase-shifting process of *Per1-luc* expression. (**a**) Phase-shifting responses to light pulses in the spontaneous locomotor activity rhythms of *Per1-luc* mice. Activity records are expressed in black histograms of activity counts in 5 min bin and double-plotted so that 48 h are shown on the *x*-axis and consecutive days on the *y*-axis. Times of activity are indicated by dark regions. A yellow square shows the time zone when light pulse was exposed (CT 12–20). Red broken lines show the regression lines fitted to the onset of activity time before and after the light exposure. (**b**) Real-time imaging of *Per1* expression in six areas of *Per1-luc* mice (mouse1) before and after an 8-h light pulse exposure. Green columns represent the time when light pulse were exposed. Red dash-and-dotted lines represent the peak time of *Per1* expression before 8-h light pulse at day 4. *indicates the peak time of *Per1-luc* expression in each day. ^#^The secondary peak at day 7 is indicated. Spontaneous locomotor activities are shown by grey histograms (counts per 5 min). Luciferin was supplied by an iPRECIO pump (15 μl h^−1^ for animals). (**c**) Peak time in *Per1* expression before and after an 8-h light pulse exposure. The green horizontal bar shows the day (day 5) when 8-h light pulse was given to mice (mouse 1, mouse 2, mouse 3). Activity records between day 4 and day 9 were analysed for mouse 1 and mouse 2, and those between day 3 and day 8 were analysed for mouse 3.

The effects of a single light pulse on circadian *Per1-luc* expression rhythms were examined in all 6 ROIs. The mice were exposed to a light pulse between circadian time (CT) 12 and CT 20, where CT 12 was defined as the time of onset of activity (Supplementary Fig. 10). An 8-h light pulse produced a large and immediate phase-shift in locomotor activity rhythm (−5.0±0.3 h, *n*=8; Fig. 6a)^28^. The phase-shift was almost complete on the day following the light pulse (1.6±0.7 days). The circadian *Per1* rhythms in the 6 ROIs were also phase-delayed but the degree of phase-shift was different among the ROIs. The circadian peak in the OB shifted the day following the light pulse (−5.0±0.5 h, *n*=3, Fig. 6b,c) and did not shift further on the following days. Conversely, the circadian peaks in the other ROIs did not shift at all on the day following the light pulse. Phase-delay shifts were detected on the second day after the light pulse with a small secondary peak between the first and second circadian peaks after the light pulse observed in all 5 ROIs (skin, 5.8±0.7 h; left ear, 6.0±1.0 h; right ear, 5.0±1.0 h; left cortex, 5.5±0.5 h; right cortex, 6.0±1.0 h, *n*=3; Fig. 6b,c and Supplementary Fig. 11). Thus, the response to a single 8-h light pulse differed in the OB versus the other ROIs (skin, ears and cortices).

## Discussion

The present study describes a new technology that enables the simultaneous, continuous, real-time monitoring in 3D space of gene expression in multiple sites over a period of several days in freely moving animals. Recently, tracking techniques for small, unmarked animals using pattern-matching techniques with one camera have been used for behavioural analysis in 2 dimensions^29^,^30^. Applying pattern-matching techniques to 3D imaging in the present study enabled the successful identification and tracking of the focal point of the imaging space in the recording cage. Tracking the position of markers on an animal in 3D enabled the identification of the direction of movement of the targets, and the simultaneous quantification of gene expression in multiple areas over a long duration. The spatial resolution of the present system is 1.63 mm, meaning that two distinct spots >1.63 mm apart are discriminated. The influence of lights scattering from an adjacent target area or from each of the triangle spots is negligible, as demonstrated by a dark area clearly demarcating the two target points. The influence of scattered light has been examined^31^. The light intensity diminishes sharply with distance from an illuminating spot to background levels in several pixels (20–30 μm^3^).

The observed bioluminescent intensity changes depend on the time-of-day, the position of the target in the 3D space of the observation chamber and the angle of the CCD camera relative to the ROI. The DuFT method identifies the 3D position of a target based on the principles of stereo photography and the SICT algorithm calibrates the signal intensity in reference to a calibration table. These techniques enabled us to monitor the circadian rhythms of clock gene expression in multiple areas of an animal's body and their responses to intrinsic and extrinsic stimuli in unconstrained mice. The bioluminescence of the *Per1* reporter used in our system was sufficient for detection with an EM-CCD camera using only a 0.5-s exposure, confirming that it is possible to perform real-time analysis of the relationship between gene expression and behaviour. In the present study, only the areas on or near the body surface were selected as targets to monitor. In the future, utilization of strong tissue-specific reporters may enable the monitoring of multiple targets inside the body regardless of their location.

Previously, *in vivo* monitoring of clock gene expression in freely moving animals has been challenging^16^,^17^. The advantage of the present method over previous studies is threefold. First is the capability of quantifying the gene expression with 3D calibration, which was lacking in the previous studies. Second is the multi-region monitoring capability, enabling the analysis of relationships among tissues or cell-specific changes/responses in gene expression, as was done here (Figs 4b,c, 5b,c and 6b,c). Third advantage is the capability to quantitatively monitor behaviour in 3D space, which enables the analysis of gene expression in correlation with specific behaviours, such as drinking and eating, with the gene expression in a particular location. Thus, the present *in vivo* monitoring system represents a novel analytical tool for examining the relation between circadian rhythms in clock gene expression and physiological outputs.

The primary limitation of the present system is the luciferin delivery system, which limits the recording duration. Luciferin delivery requires connecting animals to a delivery pump (iPRECIO) with a feed line or implanting an osmotic pump. Furthermore, to estimate the transcription rate from bioluminescent intensity, the intracellular luciferin concentration needs to be sufficient to support the luciferin–luciferase reaction. We delivered luciferin at a rate that produced a constant plasma concentration >0.2 μg μl^−1^. However, further improvements in the substrate delivery systems would facilitate studies of this kind.

Using the present system, we observed robust circadian rhythmicity in *Per1* expression at six different areas on the body of freely moving mice. The circadian rhythm was not an artefact, as the system confirmed aperiodism of clock gene expression in adult *Cry1*^*−*/*−*^/*Cry2*^*−*/*−*^-*Per1-luc* mice. Interestingly, these six areas measured showed similar circadian peaks in *Per1* expression at around CT 12. Previous studies using slice cultures *in vitro* reported *Per1* gene expression rhythms that are different in different tissues^32^,^33^. The discrepancy between the present study and the previous studies is not known, but points to the possibility of effects related to the slice preparations^34^. For example, circadian oscillations in the peripheral tissues are reported to be affected by low temperatures^35^,^36^, slices prepared under cold conditions may change the phase of circadian oscillations. With respect to the circadian phase, our results on the first few days in DD are indeed consistent with the *Per1* mRNA expression rhythms *in vivo* under LD condition by *in situ* hybridization or by quantitative polymerase chain reaction studies^23^,^24^,^25^. As is well-known, *in situ* hybridization and quantitative polymerase chain reaction studies require an intermittent tissue sampling at intervals of hours, which limits time resolution and includes individual differences. In this respect, the present system, tracking a single animal continuously over time, is much more powerful than the classical methods.

In addition, we succeeded in visualizing the kinetics of differential phase-shifts of circadian rhythms in six different tissues. The observation of a small secondary peak between the first and second day in five tissues suggests that a phase-delaying light pulse produced an internal transient desynchronization among the peripheral tissues, a phenomena that is commonly observed in jet-lag, shift-work-related disease, hypoinsulinemia and diabetes^4^,^5^.

In summary, we anticipate that advances using remote tracking devices, detecting low-intensity signals, eliminating noise and making improvements in automated algorithms will be forthcoming and will be widely applied to many areas of biomedical research, as well as finding applications beyond medicine. Our technology is by no means limited to circadian-related disease and research. Nevertheless, any other applications will need to investigate the sensitivity and spatial resolution if the tissue environment or source depth differs from what has been used in the present work.

## Methods

### Animals and housing

Mice were born and reared in the Hokkaido University animal facility where environmental conditions were controlled as follows: 12 h light/12 h dark (LD) cycle with lights on from 06:00 hours to 18:00 hours, temperature (23±1 °C) and humidity (50±5%). Animals were provided with food and water *ad libitum*. We used male adult mice (5–9-month old) of C57BL/6J background carrying either a *Per1*-promoter driven firefly *luciferase* reporter gene (*Per1-luc*) or *Bmal1*-promoter driven enhanced beetle *luciferase* (*ELuc*) reporter gene. The *Per1-luc* reporter construct was as follows: a 6.7-kb region upstream of the translation-initiation codon of *mPer1* was fused to the firefly *luciferase* (*Luc*) coding region^37^. The *Bmal1-Eluc* reporter construct was as follows: an 8.1-kb region upstream of the translation-initiation codon of *Bmal1* was fused to the *ELuc* coding region^38^. *Cry1*^*−*/*−*^/*Cry2*^*−*/*−*^ mice were acquired from Tohoku University^27^, and bred with *Per1-luc* mice carrying the *Per1 luciferase* reporter. We back-crossed the mice for >7 generations and used the resulting *Cry1*^*−*/*−*^/*Cry2*^*−*/*−*^-*Per1-luc* mice for experimentation. All animal work was performed in accordance with the Guidelines for the Care and Use of Laboratory Animals at Hokkaido University with approval (#0800277) from the Committee for Animal Experimentation.

### *Ex vivo* recording of bioluminescence

*Per1-luc* and *Bmal1-ELuc* transgenic mice were killed by cervical dislocation and decapitated between 13:00 hours and 17:00 hours. Brains were rapidly removed and placed in ice-cold Hanks's balanced salt solution (pH 7.4, Sigma-Aldrich).

In the present study, we used horizontal OB slices because it was found in a previous study that *Per1* genes are highly expressed in the mitral cell layer and granular layer cells of the mouse OB^23^. The axons of mitral cells extend toward the frontal cortex area (horizontal direction). Furthermore, CCD camera images of the freely moving mouse OB were obtained on a horizontal plane. Therefore, horizontal OB slices were deemed to be optimal for *ex vivo Per1-luc* imaging in the present study.

Slices of the whole unilateral OB slices (300-μm thick) were prepared using a microslicer (Dosaka, Osaka, Japan). OB slices at the central portion of the dorso-ventral axis were explanted on a culture membrane (Millicell CM, pore size 0.4 μm, Millipore) in a 35-mm Petri dish containing DMEM (GIBCO-Invitrogen) supplemented with NaHCO_3_ (2.7 mM, Sigma-Aldrich), HEPES (10 mM, Nacalai Tesque, Japan), kanamycin (20 mg l^−1^, GIBCO), insulin (5 μg ml^−1^, Sigma-Aldrich), putrescine (100 mM, Sigma-Aldrich), human transferrin (100 mg m1^*−*1^, Sigma-Aldrich), progesterone (20 nM, Sigma-Aldrich), sodium selenite (30 nM, GIBCO) and D-luciferin potassium salt (0.1 mM, Wako Pure Chemical Industries, Japan). After sealing with Parafilm, the OB slices were incubated at 37 °C, and bioluminescence was monitored for 1 min at 10 min intervals with a dish type luminometer (AB2500 Kronos, ATTO, Japan). In horizontal brain slices of the whole OB, *Per1* and *Bmal1* expression in the OB were measured using a PMT.

### Continuous luciferin administration

Luciferin (40 mg ml^−1^) was intraperitoneally administered into *Per1-luc* mice at a controlled flow rate (1 μl h^−1^ by an osmotic pump (Alzet, Charles River, Saint Aubin Les Elbeuf, France); 10, 15 or 30 μl h^*−*1^ by iPRECIO pump (Primtec, Tokyo, Japan)). The osmotic pump was implanted into the intraperitoneal cavity. The iPRECIO pump was connected to a ‘modified free moving animal system' (complete tethering system for mice, Eicom, Shimotoba, Kyoto, Japan) which consisted of a liquid swivel (TCS1-20, 11-μl single-channel swivel, 0.65-mm connections), balance arm, crisscross mouse harness and a tube that was guided into the intraperitoneal cavity through a subcutaneous tunnel from an incision in the dorsal neck to a ventral abdomen incision (Supplementary Fig. 1d,e). After the operation, mice were placed in a cage until the start of the experiment.

### HPLC analysis for luciferin quantification

Luciferin (40 mg ml^*−*1^) was intraperitoneally administered into *Per1-luc* transgenic mice at a constant flow rate by an osmotic pump (Alzet) or iPRECIO pump (Primtec). In all, 10–15 μl of blood was collected from the tail tip and plasma was separated (Fig. 3d and Supplementary Fig. 9a). Luciferin was supplied in the morning on day 0 and sampling started at 15:00 hours on day 1 (Fig. 3d) or 11:00 hours on day 2 (Supplementary Fig. 9a). Luciferin was eluted from 5 μl plasma with 95 μl of ethanol containing 0.5% tri-*n*-butylphosphine at 70 °C for 5 min (ref. 39). The sample was centrifuged (13,000 r.p.m., 20 min) and the supernatant was filtered with a 0.45 μm membrane filter (Millipore, Billerica, MA). The filtered sample was injected into a HPLC (high-performance liquid chromatography) system. Linear gradient elution (15–40% acetonitrile/water with 0.1% TFA, 30 min, 1.0 ml min^−1^) was adopted for separation with a chiral fused-silica column, CHIRAL-CEL OD-RH (4.6 × 150 mm; Daicel Chemical Industry, Tokyo, Japan). Luciferin was detected with a fluorescence detector (excitation *λ*=330 nm, emission *λ*=530 nm; Supplementary Fig. 9b).

To examine the time-of-day effects on plasma luciferin levels using iPRECIO pump (10, 15 and 30 μl h^*−*1^), blood was collected at 4-h intervals from 11:00 hours on day 2 until 7:00 hours on day 3. Furthermore, plasma luciferin concentrations following administration at a perfusion rate of 15 μl h^*−*1^ were re-examined on days 6– and 7 at 4-h intervals.

### *In vivo* imaging system

The imaging system was set up in a custom-made imaging box (width × depth × height; 650 × 622.5 × 700 mm, respectively) with a ventilating fan and sensors for controlling the temperature and humidity (Thermo Recorder TR-72 Ui, T&D corporation, Nagano, Japan) inside a small room (width × depth × height; 2,012 × 1,712 × 2,200 mm, respectively) with controlled environmental conditions (temperature 23±1 °C, humidity 50±5%). The interior of the imaging box was coated with black materials that minimized light halation, while the front was enclosed with a black shield curtain (Supplementary Fig. 1b). Overhead lights and computer monitors were placed outside the room. The light in the room was off during imaging and the imaging box was shielded with a black curtain. In practice, the black shield curtain was not necessary for protecting penetration of lights, since the background illumination (ca. 5.0 counts per 0.5 s) was not different, regardless of the curtain.

Two EM-CCD cameras (ImagEM C9100-13, effective number of pixels 512 × 512, cell size 16 μm × 16 μm, Hamamatsu Photonics, Hamamatsu, Shizuoka, Japan) were placed on the top panel of the imaging box at about equal angles relative to a vertical line (Supplementary Fig. 1c) using an angle adjuster (as in Fig. 1a and Supplementary Fig. 1c) in a camera box (‘A' in Fig. 1a), and were fixed to the ceiling of the imaging box to observe luminescence from the animals.

A cylindrical recording cage (200 mm diameter, 60 mm in height; Supplementary Fig. 1f) with food and a water intake nozzle connected to a water bottle (Fig. 1b and Supplementary Fig. 1f) for housing an animal for several days was placed on the central stage (as shown in Fig. 1a), which was fixed to the floor. The imaging area was limited to the inner fence (115 mm × 115 mm, 30 mm in height). Two low-intensity LED light sources (<0.01 lux at the floor of the recording cage) illuminated the recording cage from the upper left and right (Fig. 1b and Supplementary Fig. 1g) for emitting fluorescence from the scintillators (BC-490 plastic scintillator casting resin, emission *λ*=425 nm, Saint-Gobain Crystals, Hiram, OH, USA) that were attached to the animals (Fig. 1c and Supplementary Fig. 1e).

We did not use emission filters. The spectra of the scintillators did not practically overlap with the bioluminescence emission spectra of *Luc* and had a limited overlapped area of ∼40 nm with that of *ELuc* (the wavelength of maximum emission; scintillators, 425 nm, *Luc*, 562 nm and *ELuc*, 540 nm). We examined the effect of the scintillators on the total intensity of emitted bioluminescence and found that the effect was negligible (<0.1%). We therefore quantified the intensity of bioluminescence without emission filters in the present study.

To precisely identify the position of the luminescent target area in the recording cage, three scintillators (2 or 3 mm in diameter) were embedded around the target (ROIs) as reference markers (Supplementary Fig. 1e). Hair on the recording areas of the head and skin was shaved (Supplementary Fig. 1e). To get enough signals from freely moving mouse, the skull around the OB and cortex area was ground with a dental dill bur to make it thinner, which increased the intensity of bioluminescence ca. 5.3 times for the cortex and ca. 3.7 times for the OB. Finally, the recording area on the head was coated with transparent dental resin. Three scintillators were attached around the OB and skin (Supplementary Fig. 1e) on the *Per1-luc*, *Bmal1-ELuc* and *Cry1*^*−*/*−*^/*Cry2*^*−*/*−*^-*Per1-luc* mice.

Ultraviolet-light-emitting diode lights (*λ*=375 nm, TUV550A-1, Takatsuki, Japan) were placed at the top of the recording box (Fig. 1b). The intensity of the ultraviolet-light-emitting diode lights was very low and regulated by a pulse controller (Taihou Densi, Sapporo, Hokkaido, Japan) so that ultraviolet-light-emitting diode-induced fluorescent intensity of the scintillator and bioluminescent intensity from the skin were similar with 0.5 or 1.0 s exposure, as recorded by the EM-CCD camera (−80 °C cooling, EM-gain=256, max amplification). Fluorescence signals from the scintillator and bioluminescent signals from the target area were obtained using a custom video capture program (Mouse Capture). Video files were saved for subsequent analysis using the ‘Mouse Tracker' program, which consists of the DuFT and SICT algorithms. Using a pattern-matching technique with model images (Fig. 2a,b and Supplementary Fig. 4), the positions of the scintillators and ROIs in the image can be automatically detected (Figs 1f and 2a,b; see the subsection ‘Determination of ROI positions' for details). This program was made with the Matrox Imaging Library (MIL version 9.0). Circles on the image (a in the Figs 1f and 4a,d and Supplementary Movies 2, 3, 4, 5) indicate the location identified by the pattern-matching program in both the left and right images. The 3D position of the target can be computed only when the target area can be located in both the left and right camera images which include both true and false pattern matching (see the next section ‘Automatic determination of the 3D position of scintillators' for details). Images were excluded from the analysis when the software failed to identify and trace the targets. After calibrating the bioluminescent intensity of the ROIs at each 3D position (see the subsection ‘Signal-intensity calibration technique' in the ‘Methods' section for details) and post-processing analysis, *Per1* or *Bmal1* expression in the OB, skin, left ear, right ear, left cortex and right cortex were quantified (see the subsection ‘Determination of ROI positions' for details). Pixel quantification analysis yielded gene expression data. Behavioural analysis was performed using 3D plot graphs. These analyses were performed in multiple areas over a long duration. One frame required only 0.5 s to obtain (except for recording the images of *Bmal1-ELuc* mice, where 1.0 s was required). Luciferin (40 mg ml^*−*1^) was supplied by an osmotic pump or iPRECIO pump.

### Automatic determination of the 3D position of scintillators

The 3D coordinates of the scintillators on the mouse in the recording cage were derived from the 2D coordinates in a pair of images (left and right) using ‘Mouse Tracker' (based on real-time tumour-tracking radiotherapy (RTRT) software)^20^,^21^. In the RTRT system, the location of a moving tumour, such as a lung cancer, can be detected every 0.03 s with an accuracy of 1 mm by tracking the position of a spherical gold surrogate marker inserted in or near the tumour using two sets of X-ray images. The tumour is irradiated only when its location corresponds to the planned position. In this system, the tracking volume (about<50 mm^3^) is very small and the tracing time is short. In the present study, to track gene expression in freely moving mice within the recording cage (<200 mm in the *x*-, *y*-axis and <60 mm in the *z*-axis) for a long duration, we replaced the gold marker and X-ray images with a scintillator and LED in DuFT for optical imaging.

The software uses a pair of transformation matrices (one each for the left and right cameras) to convert two 2D coordinates into a 3D coordinate. The matrices are used to compute a line from each camera that passes through the scintillator. The intersection point of the two lines determines the 3D coordinate. However, the two lines will often not intersect exactly due to small inaccuracies. In that case, the centre of the shortest connection between both lines is used as the scintillator position. If the length between the lines is over 1 mm, the 3D position data are discarded during post-processing.

The transformation matrices M_l_ and M_r_ are calculated as follows. Eight marker points made with a stable luminescence marker (Glowell, LUX Biotechnology), similar to the acrylic calibration cube used in the RTRT system^20^,^21^, were used to compute the transformation matrices. The points were positioned by placing a single stable luminescence marker on a rotating disc inside the recording cage (Supplementary Fig. 5a). The stable luminescence marker emits a long-lasting very stable light, generated by a chemical reaction that is produced from a gaseous tritium light source (One Glowell kit GLO-001 Green, No. X20110, Radiant flux 4, 32 pW, 11.6 × 10^6^ photons/s, luminous flux 2.14 nlm, effective wavelength=532–536 nm). We first place the disc at the lowest height of the recording stage (1,410 mm height from the bottom of recording stage as *z*=0) and the stable luminescence marker at the maximum radius (*R*=5 cm) from the centre. We then obtain four pairs of images with the disc rotated at 45°, 135°, 225° and 315°. The disc is then raised to the maximum height (40 mm; *z*=0–5) and four more pairs of images are obtained. We used the 2D coordinates of the scintillator from six of these images, along with the known 3D position of each point, to compute the transformation matrices (M_l_ and M_r_) for each camera. M_l_ and M_r_ provide the equation of a straight line passing through the scintillator (Fig. 1d), that is, [aM]=[b], where [b] is the 2D scintillator position expressed in the homogeneous coordinates (that is, the third coordinate is set to one), [a] is any position on the line (in homogeneous coordinates), and M_l_ and M_r_ are 4 × 3 matrices of rank 3. By measuring the image scintillator locations [a] with two EM-CCD cameras for six known stable luminescence marker positions [b], one obtains 12 equations with 12 unknowns and M_l_ and M_r_ can be solved. The two matrices are stored in a file that is read by our tracking software. We also tested smaller values for the radius and height of the calibration points, but found that R=5; *z*=5 yielded the best results.

To verify the accuracy of the 3D transformation matrices, additional images of the stable luminescence marker on the rotating disc were obtained every 15° (24 pairs of images). The height of the disc was raised from the bottom of the cage at 10 mm intervals to a maximum of 40 mm (5 levels), and moved from the centre to a radius of 50 mm at 10 mm intervals (5 rings) for a total of 605 pairs of images (Supplementary Fig. 5a–c). The 2D coordinates of the stable luminescence marker on each pair of images were converted to 3D using the matrices computed above and compared with the actual coordinates.

We used a Kreuznach XENON (F0.95/25 mm, C-Mount; Jos. Schneider Optische, Bad Kreuznach, Germany) lens. The lens is adapted to the visible light in the range of 400–700 nm. In this range of wavelength, the lens passes 89 and 90.0% of the bioluminescence emitted by firefly and enhanced beetle luciferase, respectively; and 82.6% of the fluorescence from the marker scintillator passes through the lens.

The lens has the focal distance around 35 cm (centre positon at *z*=2) and barrel distortion of −2.5% at the 4 corners (_Max_ distortion) of the square-shape image. The estimated decrease of light intensity due to the distortion was ca. 37% at the corners of the image. Actually, we found the intensity at the corner was ca. 60% of that in the centre. The differences of intensity due to distortions of the lens were compensated by the left and right cameras (Fig. 6d,e).

### Signal-intensity calibration technique

Luminescence is calculated from the mean pixel value in a circular area on an image from the EM-CCD cameras. The intensity of the luminescence signal varies with the position within the recording cage, depending on the distance from the camera. To calibrate the luminescence signal, we measured the mean pixel value for the stable luminescence marker at the 605 calibration positions described in the previous section. At each calibration position, 10 images were acquired from the left and right cameras (10 photos at each position, total 6,050 pairs of images). The ten images were averaged to form a standard image for each camera at each position. The process was repeated three times and the results were averaged again. The mean pixel values are shown in Supplementary Fig. 5d,e for the left (46.19±7.65 (mean±s.d.)) and right (49.34±8.28 (mean±s.d.)) cameras, respectively.

The focus of the two cameras was set at a point at the centre of the cage, *z*=2 cm (1,610 mm height from the bottom of the recording stage). The mean pixel value of the spot at this point (*x*0, *y*0, *z*2) was used as the calibration value. The ratio of the calibration value and the pixel value at each calibration point was stored in a calibration table. The calibrated pixel values are shown in Supplementary Fig. 5f. The calibrated pixel values in these figures should be constant.

The calibration points are spread on concentric rings, like the spokes of stacked wagon wheels (Supplementary Fig. 5a–c). Calibration points are spaced closer together near the centre of the cage and spread further apart near the edges. To use the calibration table for an arbitrary point in the observation cage, we first determine the eight calibration points that surround the ROI point. The distances from the ROI point to the eight calibration points are computed. The calibration values are linearly interpolated based on the computed distances. Special cases are handled when the point is near the centre (<1 cm), outside (in a corner of the cage) or above the calibration volume (near the ceiling).

### Determination of ROI positions

A number of algorithms were developed to locate the scintillator targets on the mouse and identify the associated ROIs. All configuration parameters are specified in a Windows INI format text file. This file is used as the input to the ‘Mouse Tracker' software.

In the first case, three scintillators (diameter 3 mm) are placed on the mouse skin (Figs 1f and 2a). An image of one of the scintillators (usually about 16 × 16 pixels) is used as a search model for pattern matching in all frames of the video. Three instances of the model within the source target image are searched for with MIL according to specified constraints in the INI file. If three instances of the search model cannot be found in the target image, the frame is rejected and the next frame is examined. On successful identification of three scintillators, the resulting triangle is analysed to determine if the three points are actually the three scintillators and not the result of misidentification due to image noise.

The distances between the three points are computed. If the longest distance exceeds a maximum threshold specified in the INI file, the frame is rejected. Similarly, the frame is rejected if the minimum distance is less than a specified threshold. The distance between points depends on how the scintillators are placed on the mouse skin and the distance from the camera to the mouse when the frame was recorded. The distances can be displayed with ‘Mouse Tracker' so that the user can determine reasonable values for the minimum and maximum thresholds.

The acceptance level is the value (per cent) at which the correlation (pattern match score) between the model and the pattern in the image is considered a match. A perfect match is 100% and no correlation is 0%. Because the captured video images have considerable noise, perfect matches are generally unobtainable. We found that the acceptance level should be set below the default value of 70%. If the acceptance level is set too high, MIL will not find any matches for the scintillators in the video frame, and if set at too low, the chance of false matches is increased and search time may also increase. The user must determine the optimal value for the acceptance level empirically, depending on the signal intensity of the target. We found that a value of about 30% worked well in our experiments (Supplementary Movies 2, 3, 4, 5). Note that the raw video images contain both true and false pattern matching of model images.

For analysis of skin, we place the three scintillators so as to form an approximate isosceles triangle. The triangle base is assumed to be the line between the points with greatest distance. The ROI is assumed to be at the midpoint of the base line. The ROI is a circle whose diameter is set in the INI file. The ROI circle is displayed on each frame, so that the user can adjust the values for the ROI diameter. The ROI should tightly encircle the shaved region on the mouse skin where the bioluminescent signal is to be measured. We found that an ROI diameter of 12 pixels worked well. The average value of the pixels within the ROI circle is calculated and written to the output file.

For the OB, three scintillators (diameter 2 mm) are placed on the mouse head to form an approximate equilateral triangle with the OB at the centroid (Fig. 1f). As with skin, the diameter of the ROI circle must be empirically determined by the user, by observing the circle displayed on the video. In our experiments, an ROI diameter of 9 pixels worked well. The distances between the three scintillator points are computed. If the ROI of the OB cannot be identified, the frame is discarded and no further calculations are performed.

To locate other ROIs, such as the left and right ears and cortices, we developed an alternative method using a model image of the mouse head for pattern matching (Fig. 2b and Supplementary Fig. 4). A representative image of the mouse head is captured and stored in a file. This image is used as a search model for pattern matching in all frames of the video with rotated versions of the model (Fig. 2b). Note that rotation of the head model significantly increases search time. More than one model head image can be specified to increase the chance of successful matches. Mouse ear ROIs (20 pixels) and cortex ROIs (8 pixels) are defined relative to the top-left corner of the model head image (Fig. 2b), specified in the INI file. If the mouse model head image is found within a video frame, the OB can also be located by searching for the three small scintillators on the head within the region in which the model head image was found. This decreases the chance of mismatches. Three instances of the model scintillators within the source target image are searched as described above.

If an ROI can be successfully located in both the left and right images, the 3D coordinates of the ROI can be computed. If the 3D ROI coordinates indicate an unreasonable position (that is, outside the observation cage), due to pattern mismatching, then the frame is discarded. Otherwise, the pixel values within the ROI circle of specified diameter (OB 9 pixels; Skin12 pixels; ear 20 pixels; cortex 8 pixels) in the left and right images are averaged. The 3D coordinates are used to calibrate the raw pixel averages with a pre-computed calibration table. Average calibrated bioluminescent intensity from the left and right images was saved in a ‘.csv' file.

After analysis by ‘Mouse Tracker', the data in the ‘.csv' file was analysed in a post-processing step. If the size of the triangle exceeds thresholds (the triangle area with three points between minimum 100–200 pixels and maximum 700–900 pixels in the OB; minimum 500–600 pixels and maximum 1,000–1,200 pixels in the skin), the frame is discarded. The distances between the three scintillator points is also checked. The remaining data was checked for the distance between the head and skin regions. If the distance exceeds a maximum threshold (default 80 mm), the frame is rejected. Similarly, the frame is rejected if the distance is less than a threshold (default 20 mm). Finally, gene expression in the ROI is quantified (mean pixel value) for each recording session (Supplementary Fig. 6).

Although many frames are discarded with this method, a sufficient number of good frames were found to enable the recording of the rhythmic expression of target genes (Supplementary Fig. 7a). We used the frames in which the mouse was not moving much (Supplementary Fig. 8).

The major sources of error in estimation of 3D position are twofold; the error of positioning in the measurement box and the error of pattern matching for the marker (triangle shape). On the basis of these errors, spatial resolution was calculated to be 1.63 mm.

To calculate the 3D-positioning error, we assessed the 3D-positioning of the target area within the recording cage using the apparatus shown in Fig. 1a and based on the 605 marker points shown in Supplementary Fig. 5. From the 2D coordinates of the scintillators in each camera's image, two straight lines passing through a scintillator were calculated and the 3D coordinates of the scintillator were determined by calculating the intersection of these two lines (Fig. 1d). In most cases, the two lines will not intersect exactly due to small inaccuracies. We calculated the error determining intersection points by the 3D transformation matrices with DuFT.

### Phase-shifting responses to light pulses

*Per1-luc* transgenic mice were maintained for at least 1 week after surgery in a LD cycle. Recording of *Per1*-*luc* expression started after transferring to DD. Mice were exposed to an 8-h light pulse at a specified CT (CT 12) on day 5 in DD. Recording was discontinued during the 8-h light exposure and resumed thereafter (Supplementary Fig. 10a). The activity onset time was designated as CT 12. For long recording, we obtained images for 10 min, at 0.5 s per frame for *Per1-luc* within each 30 min interval. Locomotor activity rhythm was monitored using an on-line PC (CLOCKLAB, Actimetrics) and spontaneous locomotor activity was recorded using an infrared sensor. Daily activity onset was visually estimated from standard double-plot actograms of locomotor activity behaviour. The phase-shift in locomotor activity rhythm in response to the 8-h light pulse was determined for several days preceding and following the day of the light pulse. A line was manually positioned through times of activity onset over several days (Fig. 6a). The primary peak time of *Per1-luc* expression was determined as the time of highest intensity of *Per1-luc* expression around a continuous high-intensity area during each circadian cycle (Fig. 6b and Supplementary Fig. 11).

### Circadian rhythmicity analysis of *Per1* expression *in vivo*

Circadian rhythmicity of quantified *Per1* expression was analysed using the Cosinor program (Cosiner.exe, version 2.3; Circadian Rhythm Laboratory). This program uses a least-squares regression method to fit a cosine wave to a time series and identify the best-fit cosine wave with period information. In Supplementary Table 1, a period was determined by performing cosinor analysis between 23.5 h and 24.0 h with 0.1 h step. This approach consists of minimizing the sum of squared deviations between the data and the fitted cosine curve. The larger this residual sum of squares is, the greater the uncertainty of the estimated parameters is. Best fitting is the percentage of variance divided by the total variance (and multiplied by 100).

The significance of the rhythm is calculated using the zero amplitude test, in which the null hypothesis is that amplitude is zero. Rejection of this null hypothesis signifies that the fitted curve (implying rhythmicity) approximates the data more closely than does a straight line of zero slope (implying constancy).

### Data availability

We declare that all data supporting the findings of this study are available within the article and its Supplementary Information files.

## Additional information

**How to cite this article:** Hamada, T. *et al*. *In vivo* imaging of clock gene expression in multiple tissues of freely moving mice. *Nat. Commun.* 7:11705 doi: 10.1038/ncomms11705 (2016).

## Supplementary Material

Supplementary Information Supplementary Figures 1-11 and Supplementary Tables 1-3.

Supplementary Movie 1 Tracking of a scintillator placed on the olfactory bulb

Supplementary Movie 2 Tracking of Per1-luc bioluminescence in 2 regions at subjective night

Supplementary Movie 3 Tracking of Per1-luc bioluminescence in 2 regions at subjective day

Supplementary Movie 4 Tracking of Per1-luc bioluminescence in 6 regions of a wild-type mouse

Supplementary Movie 5 Tracking of Per1-luc bioluminescence in 6 regions of a Cry1-/-/Cry2-/- mouse

## References

[b1] Young M. W. & Kay S. A. Time zones: a comparative genetics of circadian clocks. Nat. Rev. Genet. 2, 702–715 (2001). 11533719 10.1038/35088576

[b2] Tei H. . Circadian oscillation of a mammalian homologue of the *Drosophila* period gene. Nature 389, 512–516 (1997). 9333243 10.1038/39086

[b3] Sun Z. S. . RIGUI, a putative mammalian ortholog of the *Drosophila* period gene. Cell 90, 1003–1011 (1997). 9323128 10.1016/s0092-8674(00)80366-9

[b4] Mahoney M. M. Shift work, jet lag, and female reproduction. Int. J. Endocrinol. 2010, 813764 (2010). 20224815 10.1155/2010/813764 PMC2834958

[b5] Marcheva B. . Disruption of the clock components CLOCK and BMAL1 leads to hypoinsulinaemia and diabetes. Nature 466, 627–631 (2010). 20562852 10.1038/nature09253 PMC2920067

[b6] Zhang W. . Rapid *in vivo* functional analysis of transgenes in mice using whole body imaging of luciferase expression. Transgenic Res. 10, 423–434 (2001). 11708652 10.1023/a:1012042506002

[b7] Abraham U., Prior J. L., Granados-Fuentes D., Piwnica-Worms D. R. & Herzog E. D. Independent circadian oscillations of Period1 in specific brain areas *in vivo* and *in vitro*. J. Neurosci. 25, 8620–8626 (2005). 16177029 10.1523/JNEUROSCI.2225-05.2005 PMC6725522

[b8] Tahara Y. . *In vivo* monitoring of peripheral circadian clocks in the mouse. Curr Biol. 22, 1029–1034 (2012). 22578421 10.1016/j.cub.2012.04.009

[b9] Sakamoto A. . Influence of inhalation anesthesia assessed by comprehensive gene expression profiling. Gene 356, 39–48 (2005). 15967596 10.1016/j.gene.2005.03.022

[b10] Bellet M. M. . Ketamine influences CLOCK:BMAL1 function leading to altered circadian gene expression. PLoS ONE 6, e23982 (2011). 21887357 10.1371/journal.pone.0023982 PMC3161090

[b11] Ohe Y., Iijima N., Kadota K., Sakamoto A. & Ozawa H. The general anesthetic sevoflurane affects the expression of clock gene mPer2 accompanying the change of NAD+ level in the suprachiasmatic nucleus of mice. Neurosci Lett. 490, 231–236 (2011). 21195744 10.1016/j.neulet.2010.12.059

[b12] Kadota K. . Time-dependent repression of m*Per2* expression in the suprachiasmatic nucleus by inhalation anesthesia with sevoflurane. Neurosci Lett. 528, 153–158 (2012). 22902991 10.1016/j.neulet.2012.07.061

[b13] Cheeseman J. F. . Generl anesthesia alters time perception by phase shifting the circadian clock. Proc. Natl Acad. Sci. USA 109, 7061–7066 (2013). 10.1073/pnas.1201734109 PMC3344952 22509009

[b14] Anzai M. . Direct and specific effect of sevoflurane anesthesia on rat Per2 expression in the suprachiasmatic nucleus. PLoS ONE 8, e59454 (2013). 23555676 10.1371/journal.pone.0059454 PMC3605447

[b15] Mori K. . Epigenetic suppression of mouse *Per2* expression in the suprachiasmatic nucleus by the inhalational anesthetic, sevoflurane. PLoS ONE 9, e87319 (2014). 24498074 10.1371/journal.pone.0087319 PMC3909093

[b16] Saini C. . Real-time recording of circadian liver gene expression in freely moving mice reveals the phase-setting behavior of hepatocyte clocks. Genes Dev. 27, 1526–1536 (2013). 23824542 10.1101/gad.221374.113 PMC3713432

[b17] Yamaguchi S. . View of a mouse clock gene ticking. Nature 409, 684 (2001). 11217850 10.1038/35055628

[b18] Tanaka K. F. . Expanding the repertoire of optogenetically targeted cells with an enhanced gene expression system. Cell Rep. 2, 397–406 (2012). 22854021 10.1016/j.celrep.2012.06.011

[b19] Coxeter H. S. M. Projective Geometry 2nd edn Springer-Verlag (1987).

[b20] Shirato H., Shimizu S., Shimizu T., Nishioka T. & Miyasaka K. Real-time tumour-tracking radiotherapy. Lancet 353, 1331–1332 (1999). 10.1016/S0140-6736(99)00700-X 10218540

[b21] Shirato H. . Physical aspects of a real-time tumor-tracking system for gated radiotherapy. Int. J. Radiat. Oncol. Biol. Phys. 48, 1187–1195 (2000). 11072178 10.1016/s0360-3016(00)00748-3

[b22] Otsu N. A threshold selection method from gray-level histograms. IEEE Trans. Syst. Man. Cybernet 9, 62–66 (1979).

[b23] Hamada T., Honma S. & Honma K. Light responsiveness of clock genes, Per1 and Per2, in the olfactory bulb of mice. Biochem. Biophys. Res. Commun. 409, 727–731 (2011). 21624349 10.1016/j.bbrc.2011.05.076

[b24] Tanioka M. . Molecular clocks in mouse skin. J. Invest. Dermatol. 129, 1225–1231 (2009). 19037239 10.1038/jid.2008.345

[b25] Wakamatsu H. . Restricted-feeding-induced anticipatory activity rhythm is associated with a phase-shift of the expression of mPer1 and mPer2 mRNA in the cerebral cortex and hippocampus but not in the suprachiasmatic nucleus of mice. Eur. J. Neurosci. 13, 1190–1196 (2001). 11285016 10.1046/j.0953-816x.2001.01483.x

[b26] Nelson W., Tong Y. L., Lee J. K. & Halberg F. Methods for cosinor-rhythmometry. Chronobiologia 6, 305–323 (1979). 548245

[b27] van der Horst G. T. . Mammalian Cry1 and Cry2 are essential for maintenance of circadian rhythms. Nature 398, 627–630 (1999). 10217146 10.1038/19323

[b28] Beersma . The progression of circadian phase during light exposure in animals and humans. J. Biol. Rhythms 24, 153–160 (2009). 19346452 10.1177/0748730408330196

[b29] de Chaumont F. . Computerized video analysis of social interactions in mice. Nat. Methods 9, 410–417 (2012). 22388289 10.1038/nmeth.1924

[b30] Pérez-Escudero A., Vicente-Page J., Hinz R. C., Arganda S. & de Polavieja G. G. idTracker: tracking individuals in a group by automatic identification of unmarked animals. Nat. Methods 11, 743–748 (2014). 24880877 10.1038/nmeth.2994

[b31] Yoshikawa T. . Spatiotemporal profiles of arginine vasopressin transcription in cultured suprachiasmatic nucleus. Eur. J. Neurosci. 42, 2678–2689 (2015). 26342201 10.1111/ejn.13061

[b32] Abe M. . Circadian rhythms in isolated brain regions. J. Neurosci. 22, 350–356 (2002). 11756518 10.1523/JNEUROSCI.22-01-00350.2002 PMC6757616

[b33] Yamazaki S. . Resetting central and peripheral circadian oscillators in transgenic rats. Science 288, 682–685 (2000). 10784453 10.1126/science.288.5466.682

[b34] Okada M., Sakaguchi T. & Kawasak,i K. Correlation between anti-ubiquitin immunoreactivity and region-specific neuronal death in N-methyl-D-aspartate-treated rat hippocampal organotypic cultures. Neurosci Res. 22, 359–366 (1995). 7478300 10.1016/0168-0102(95)00911-c

[b35] Morf J. . Cold-inducible RNA-binding protein modulates circadian gene expression posttranscriptionally. Science 338, 379–383 (2012). 22923437 10.1126/science.1217726

[b36] Buhr E. D., Yoo S. H. & Takahashi J. S. Temperature as a universal resetting cue for mammalian circadian oscillators. Science 330, 379–385 (2010). 20947768 10.1126/science.1195262 PMC3625727

[b37] Inagaki N., Honma S., Ono D., Tanahashi Y. & Honma K. Separate oscillating cell groups in mouse suprachiasmatic nucleus couple photoperiodically to the onset and end of daily activity. Proc. Natl Acad. Sci. USA 104, 7664–7669 (2007). 17463091 10.1073/pnas.0607713104 PMC1857228

[b38] Nakajima Y. . Enhanced beetle luciferase for high-resolution bioluminescence imaging. PLoS ONE 5, e10011 (2010). 20368807 10.1371/journal.pone.0010011 PMC2848861

[b39] Niwa K., Nakamura M. & Ohmiya Y. Stereoisomeric bio-inversion key to biosynthesis of firefly D-luciferin. FEBS Lett. 580, 5283–5287 (2006). 16979628 10.1016/j.febslet.2006.08.073

