# Supplementary material for: In vivo imaging of clock gene expression in multiple tissues of freely moving mice
Source: Nat Commun. 2016 Jun 10;7:11705. doi: 10.1038/ncomms11705 (PMC5446038; doi:10.1038/ncomms11705)
Supplement: Supplementary Information — Supplementary Figures 1-11 and Supplementary Tables 1-3. [file ncomms11705-s1.pdf]

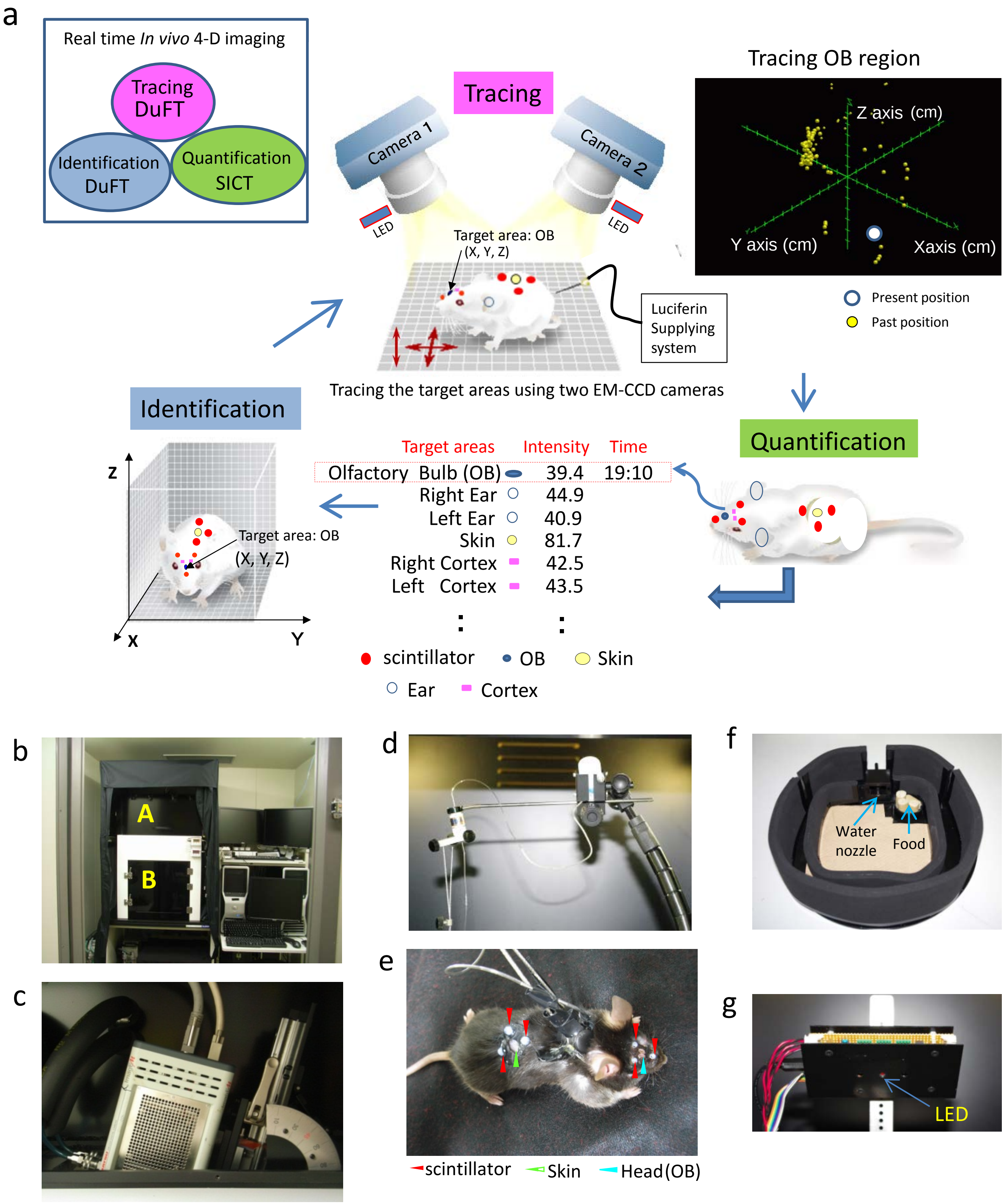

Supplementary Figure 1a-g

Supplementary Figure 1

***In vivo* imaging system in freely moving mouse**

(**a**) Scheme of *in vivo* imaging system. The system performed by “Mouse Tracker” consists of DuFT (identification, tracing in the ROI) and SICT (quantification of the gene expression in the ROI). The schematic figure shows an example of tracing olfactory bulb (OB). First, scintillators on the mouse head are illuminated with LED light. The 3D position of the OB is automatically located by DuFT (pattern match program). Next the position of the OB in the cage is traced by DuFT (tracing program). Calculating the present position of the OB, we get the X, Y, Z position. Using this position data, we calibrate the intensity of bioluminescence in the OB by SICT. This process is performed simultaneously in multi-areas and continues for a long time. (**b**) The upper photo shows the camera box (A), imaging box (B) and recording computer in the constant temperature and humidity controlled room. (**c)** High-sensitive EM-CCD camera with angle adjuster in the dark box. (**d**) Luciferin administration system. The iPRECIO pump was connected to a liquid swivel for freely moving recording. (**e**) Mouse attached with scintillator and tube connected luciferin supplying iPRECIO pump through liquid swivel. Head and skin hair were shaved. (**f**) Recoding cage (200 mm diameter). The photo shows the food pellet in the upper right side and water intake nozzle connected with a water bottle in the middle top. (**g**) UV-LED light stand (Max wave length, λ =375 nm). UV-LED light is controlled by a pulse control program.

a

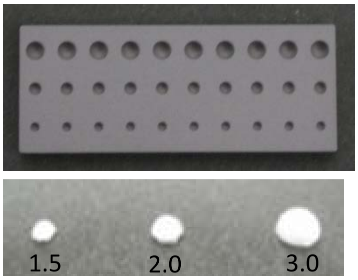

b

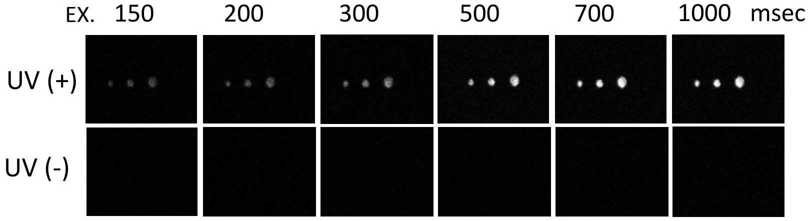

c

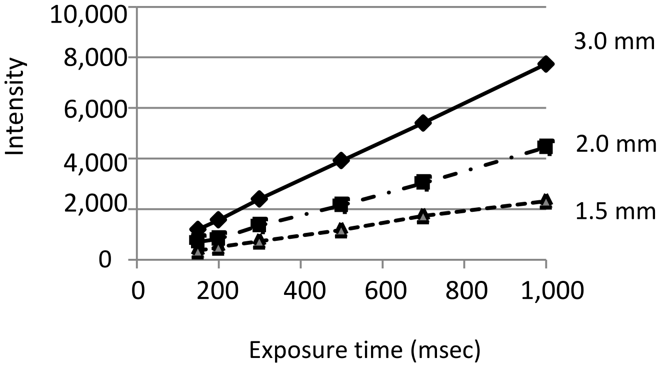

Supplementary Figure 2a-c

Supplementary Figure 2

Scintillator reaction with LED light

(**a**) Scintillator mold (upper photo). Three different diameters of scintillators (lower photo) in shape of a half sphere were made. We used 2 mm diameter for head maker and 3 mm diameter for skin marker. (**b**) The fluorescence of three scintillators (diameter 1.5, 2, 3 mm) by LED light (Max wave length, λ=375 nm). Fluorescent intensities were increase with LED light exposure time. UV (+): LED light exposure. UV (-): LED light No exposure. (**c**) The relationship between the intensity of scintillator and the exposure time of EM-CCD camera. The UV-LED of weak and stable intensity was controlled by UV-light control program to be almost the same level with bioluminescent intensity from the skin with 0.5 or 1.0 sec exposure time recording.

Per1 transgenic mouse (B/6 background)
Recording regions

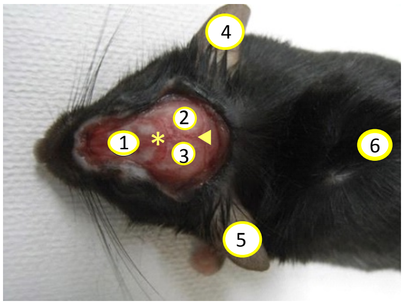

1. Olfactory bulbs (OB)
2. Right cortex
3. Left cortex
4. Right ear
5. Left ear
6. Skin

Supplementary Figure 3

Supplementary Figure 3

Recording regions of *Per1* gene expression

*. ▲ indicate the region of the bregma and lambda, respectively. The skull above the OB, left cortex and right cortex was grounded by dental drill bur to detect enough bioluminescent signals from the target area.

Model head image for pattern matching (OB)

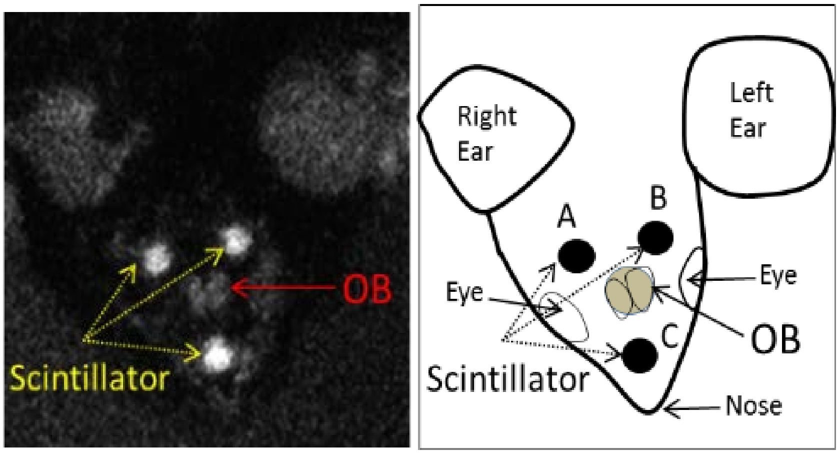

● A,B,C; Scintillator (diameter 2 mm)

Supplementary Figure 4

Supplementary Figure 4
Model head image for pattern matching of OB

a

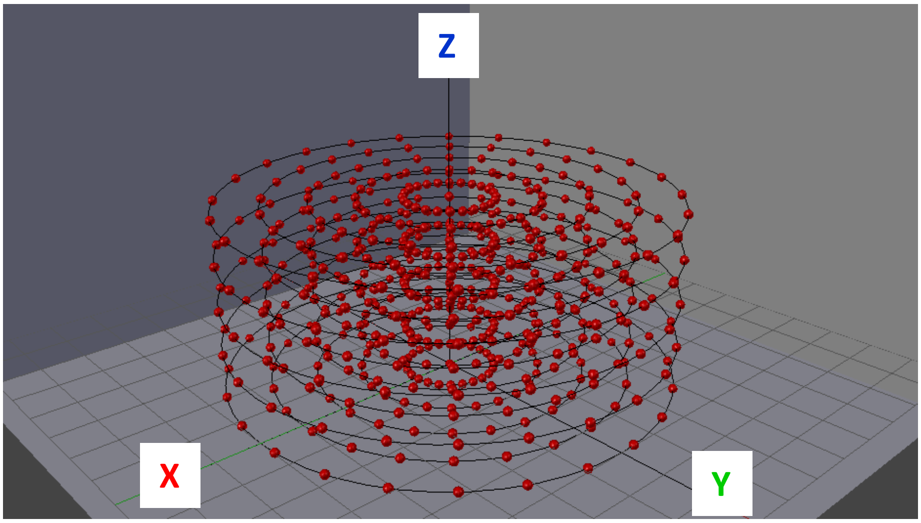

b

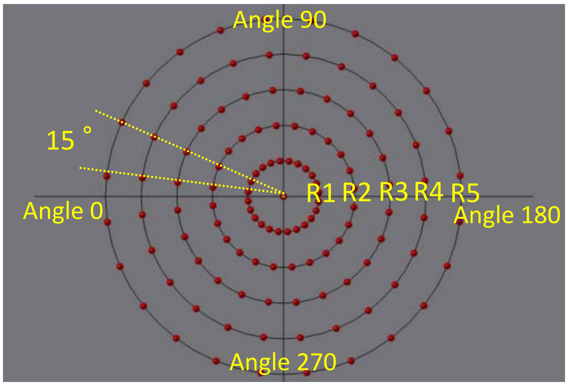

c

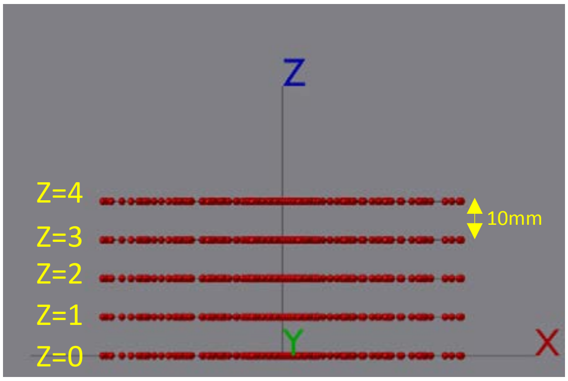

Supplementary Figure 5a-c

d

Left

Left=46.19± 7.65 (Left camera; Mean ± S.D.M.)

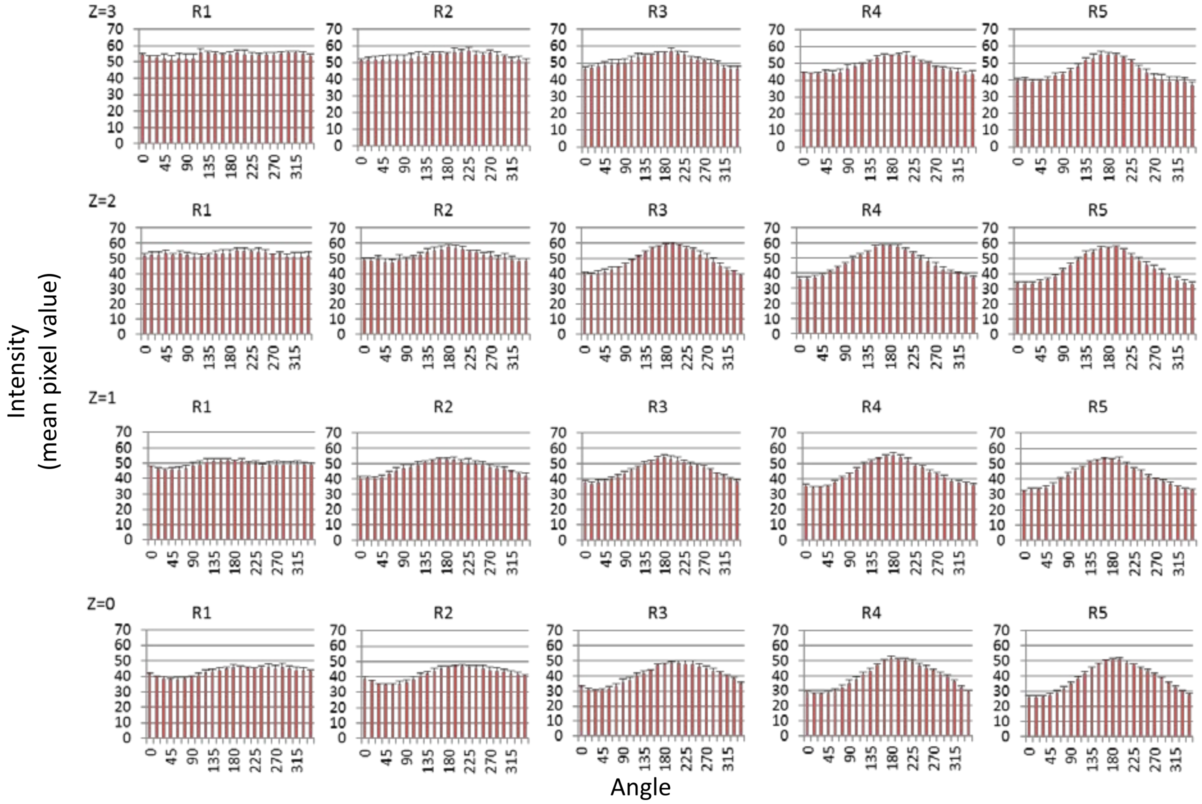

e

Right

Right=49.34 ±8.28 (Right camera; Mean ± S.D.M)

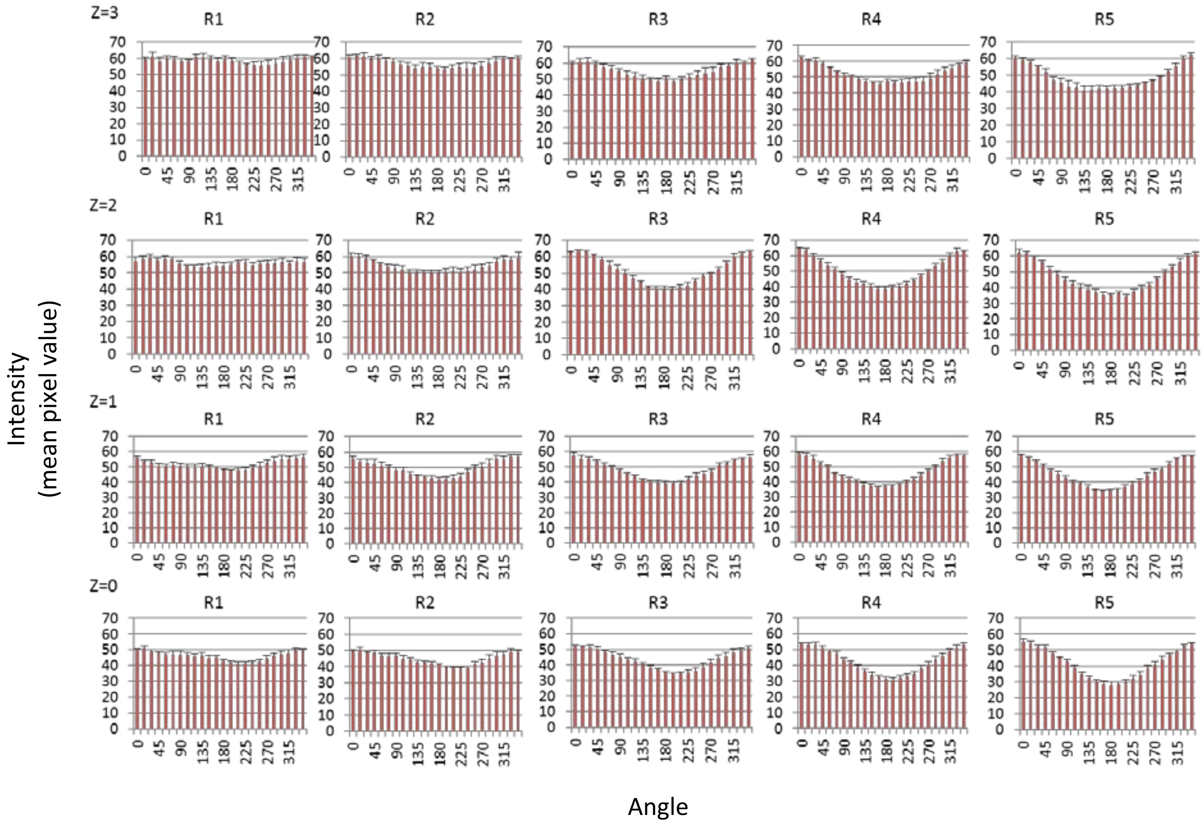

Supplementary Figure 5d-e

f

Mean Intensity, 54.79± 0.16
(Left +Right/2; Mean ± S.D.M)

XYZ 0,0,2 center; 54.69± 1.55

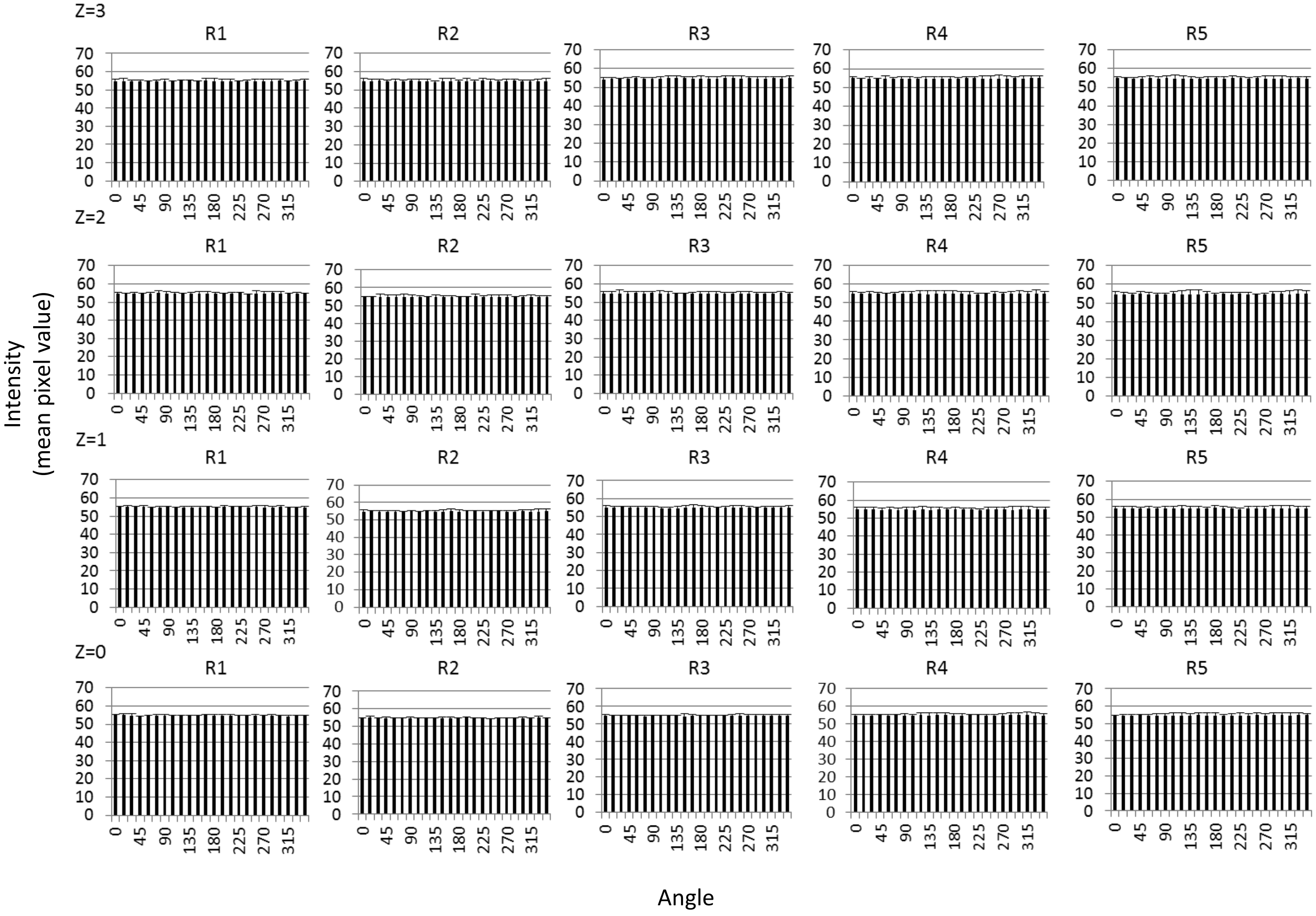

Supplementary Figure 5f

Supplementary Figure 5

Calibration of luminescence within the recording cage

605 calibration positions (including 5 center points) of stable luminescence marker for 3D transformation matrices and calibration of the luminescence signal are shown in (**a)**. A view of calibration positions from the top in (**b**) and that from the side (**c**). (**d, e**) The luminescence intensity before calibration within the recording cage. The average pixel value of stable luminescence marker are shown in (**d**) and (**e**) for the left and right cameras, respectively. Representative data show the mean differences from a pair of images from Z=0 ~ Z=3 with 3 trials. The luminescence intensity increases close to the EM-CCD camera and decreases far from the EM-CCD camera. (**f**) The calibrated pixel values from the data in (**d**) and (**e**) using the calibration table. The standard deviation of the calibrated pixel values is 0.16 (calibrated mean intensity is 54.79 ± 0.16, the intensity at the center (X0, Y0, Z2) position is 54.69 ± 1.55).

Quantificaiton of gene expressions in multiple area of the head and skin

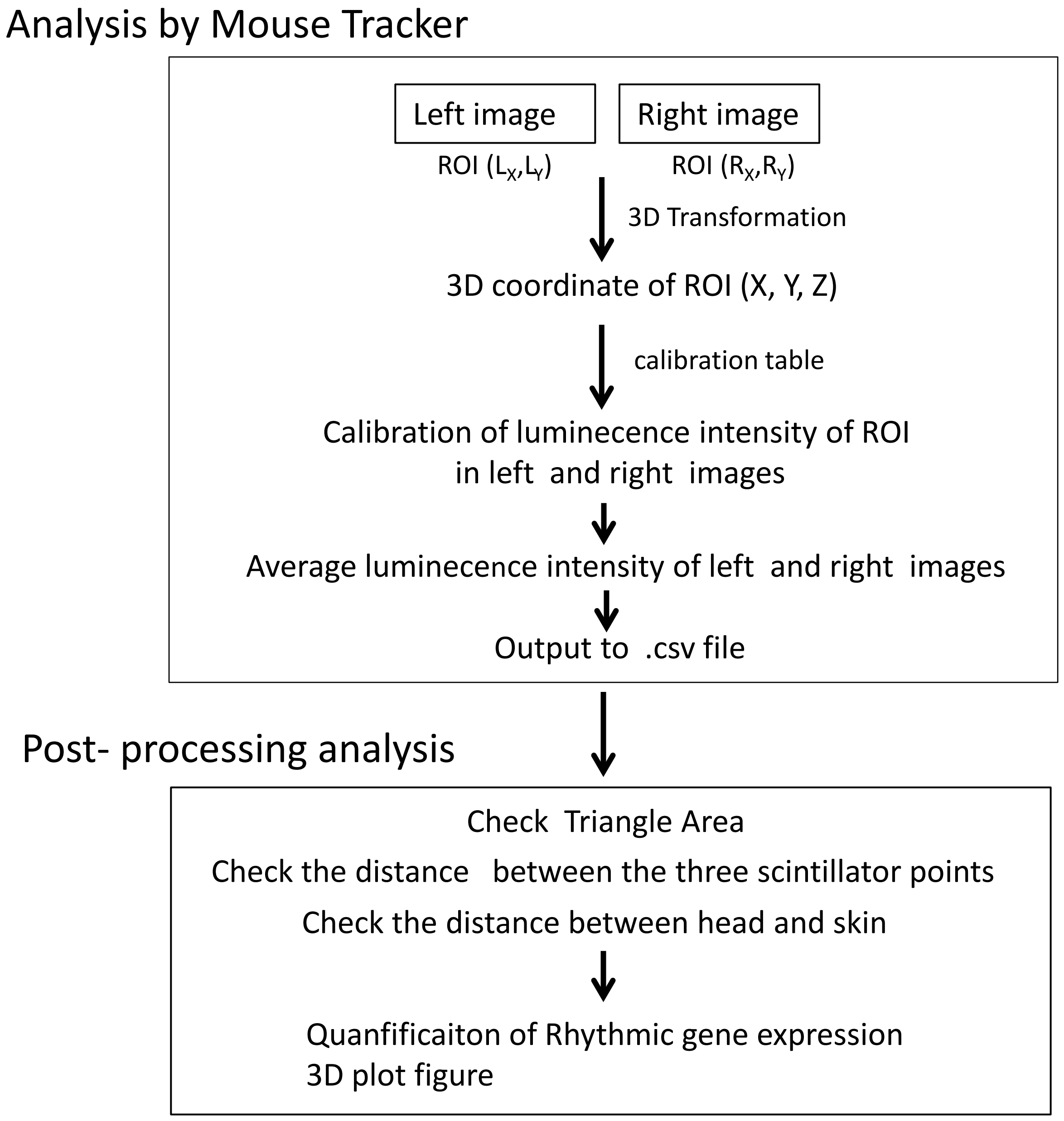

Supplementary Figure 6

Supplementary Figure 6

Flowchart of quantification of bioluminescent intensity in the ROI

First, the data of left and right images were analyzed by “Mouse Tracker”. 3D coordinate of ROI as shown in figure 2 (the frame of 3D position error were discard, see Methods for details), is calibrated referring to the calibration table. Next, averaged bioluminescent intensity data of left and right images are outputted in csv files. The csv files are analyzed by post-processing program which consisted of the system checking triangle area, the distances between the three scintillator points and the distance between OB and skin etc (see Methods for details). Finally, the bioluminescent intensity of ROI in freely moving mice is quantified and analyzed with gene expression. The 3D coordinate during each recording units are used for making 3D plot figure.

a

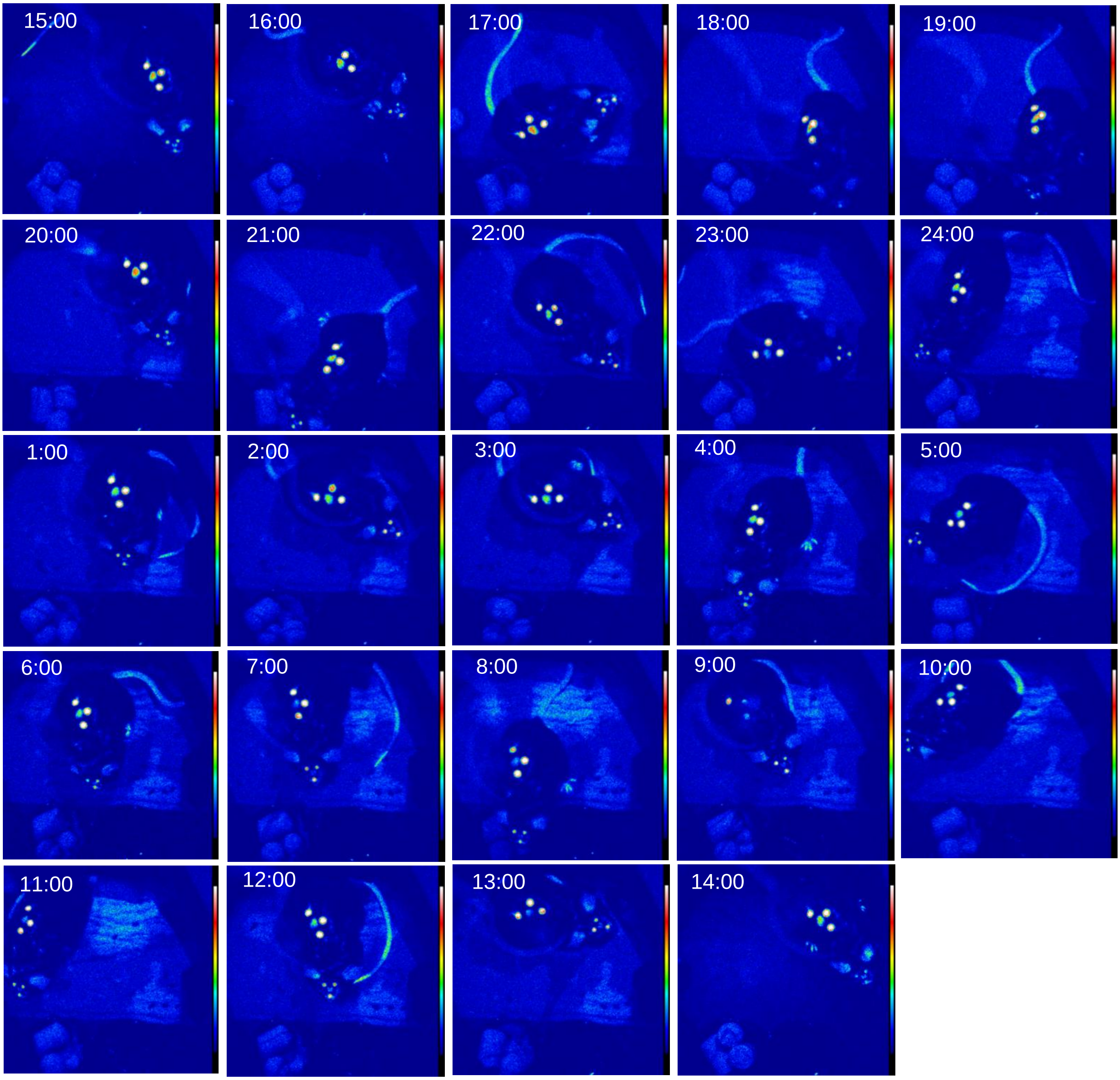

Supplementary Figure. 7a

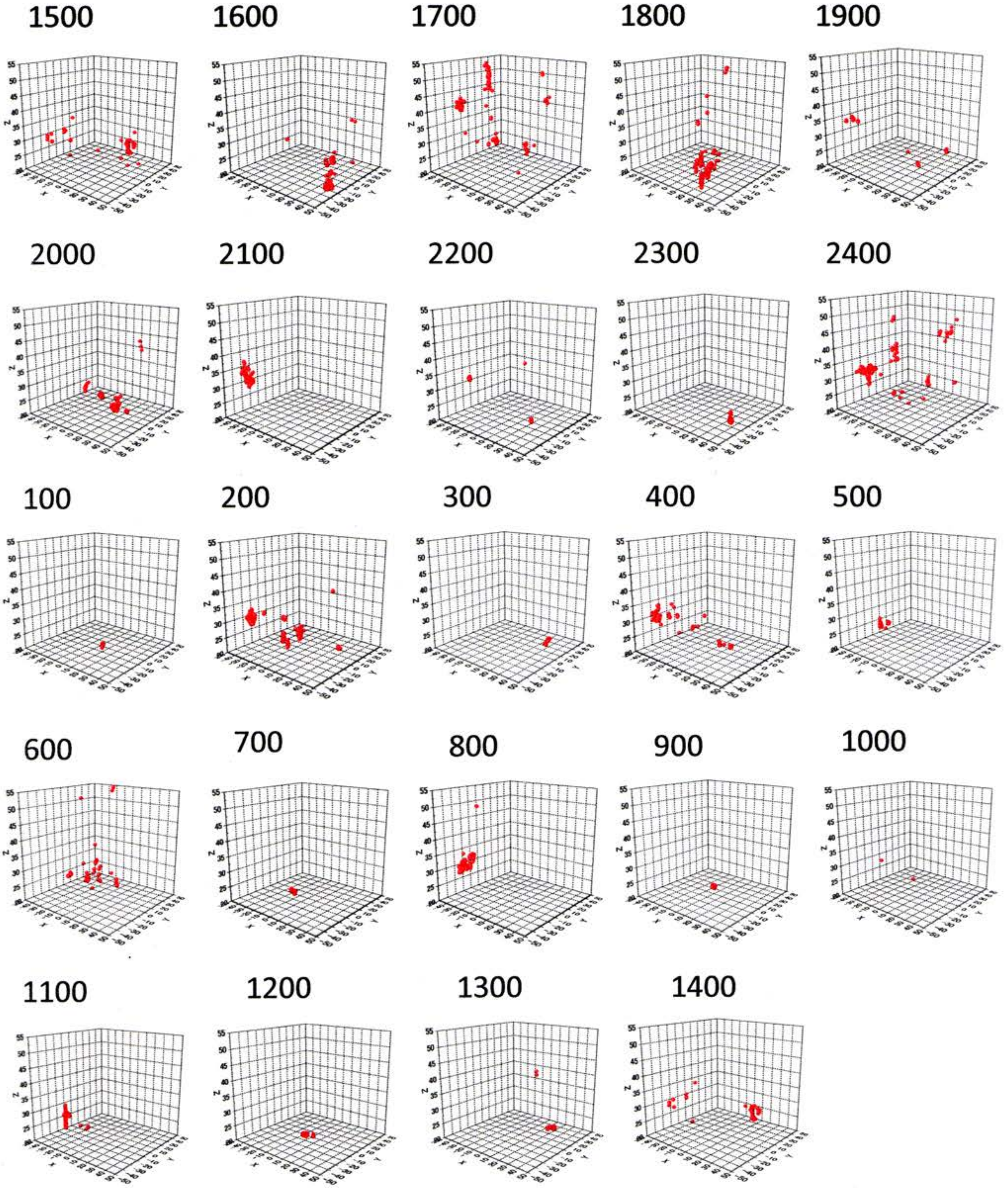

Supplementary Figure. 7b

Supplementary Figure 7

Long recording the *Per1* gene expression

(**a**) Representative images of *Per1-luc* mice at each recording time. Luciferin (40 mg ml^{-1}) was applied to the intraperitoneal cavity by iPRECIO pump (15 µl hr^{-1}). (**b**) 3D plot figures tracing the OB in (**a**) at each recording time (10 min recording) in freely moving *Per1-luc* mice. Mice move around actively during night time and are quiet during daytime.

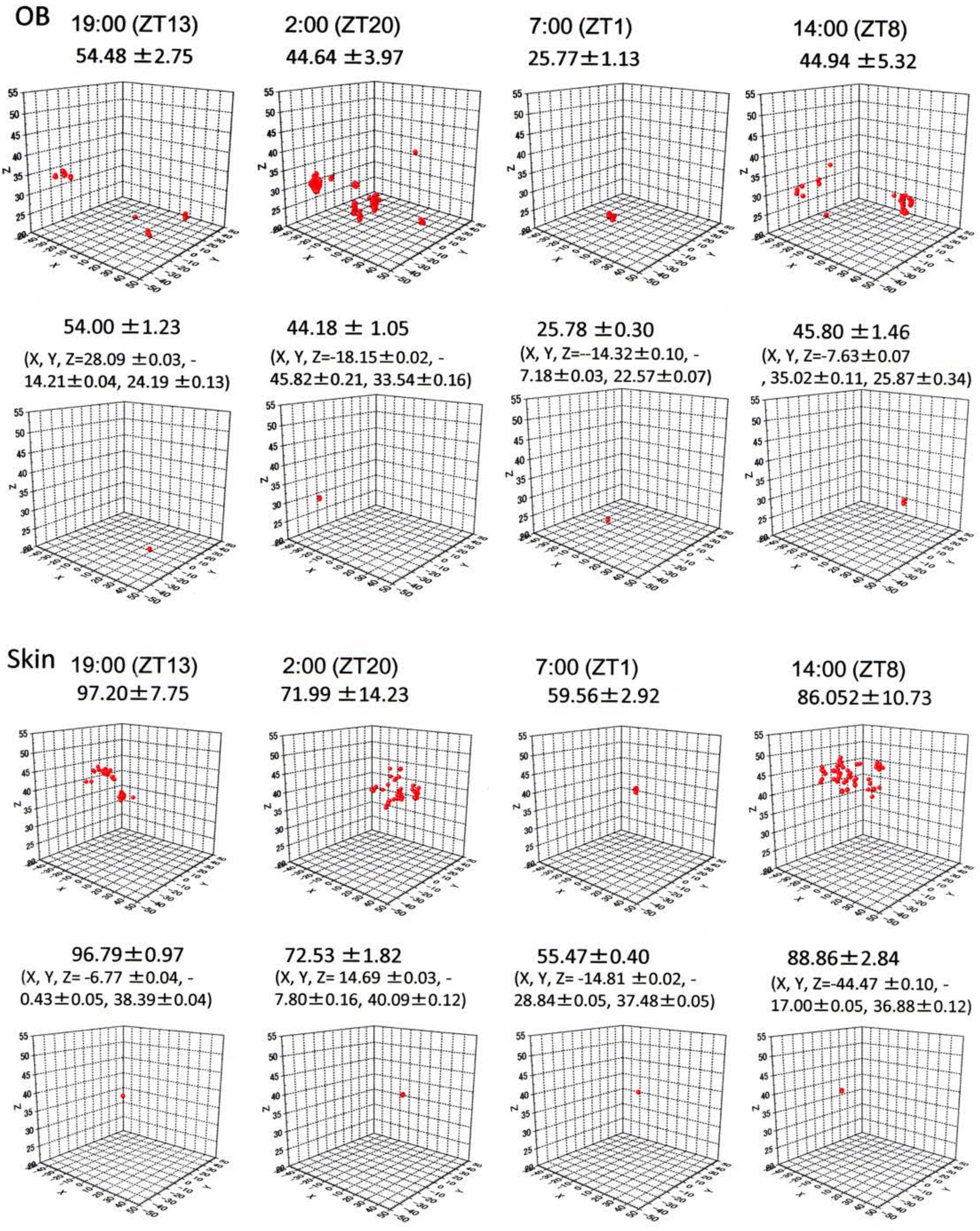

Supplementary Figure 8

Supplementary Figure 8

Calibrated intensity of bioluminescence of *Per1-luc* expression

We selected the images in which mice were not moving {the differences of 3D coordinates (X, Y, Z) are within 1 mm} for several sec, and compared with that of 10 min recording. Upper figures in the OB and skin show calibrated intensity (mean ± s.d.) for 10 min and the 3D plot figure recording. Lower figures in the OB and skin show calibrated intensity (mean ± s.d.) for 10 sec which is stable with moving distance within 1 mm (X, Y, Z) during 10 min and the 3D plot figure recording. 3D coordinates (X, Y, Z) indicate the mean positon during 10 sec.

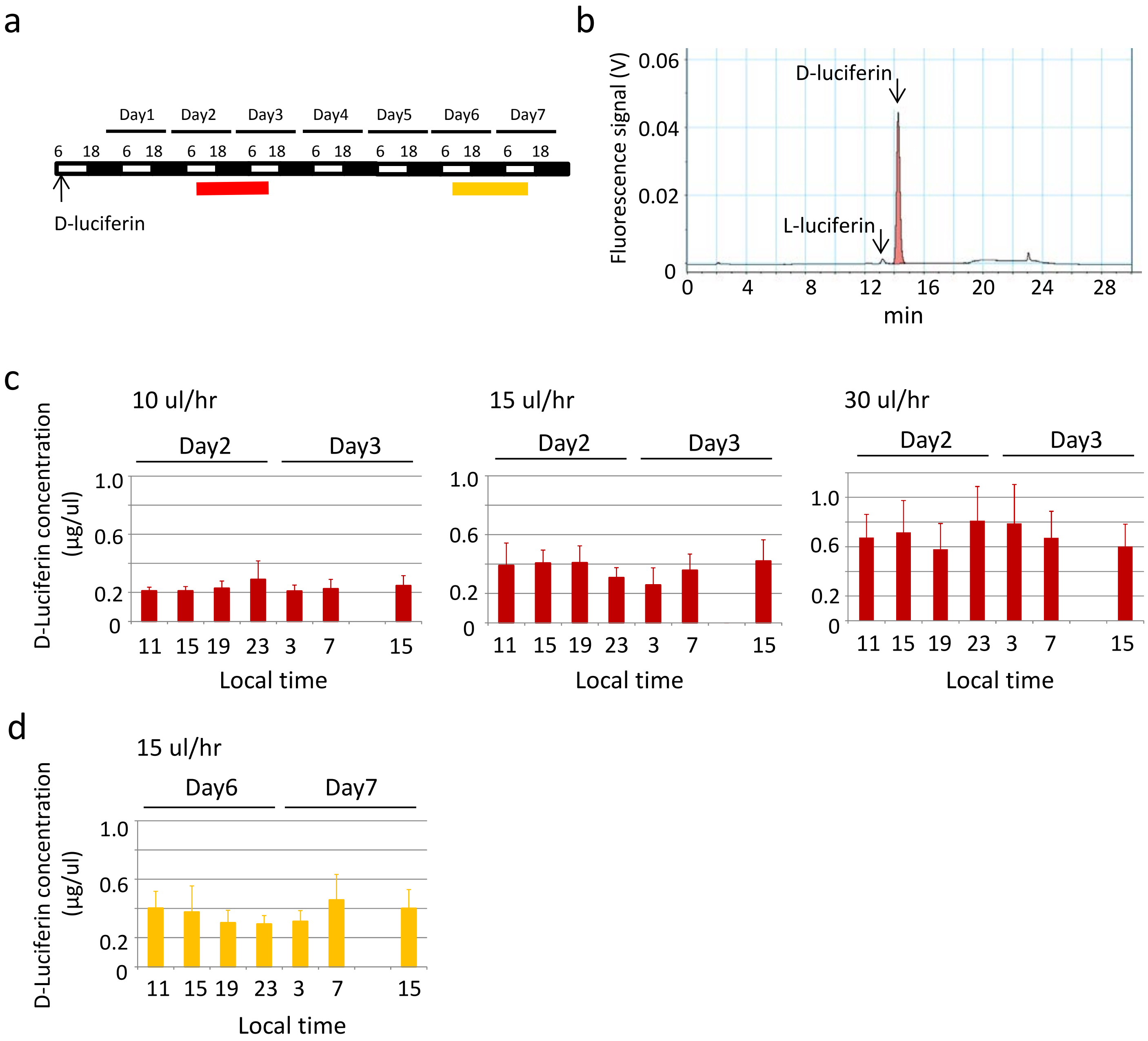

Supplementary Figure 9a-d

Supplementary Figure 9

Stably supplying luciferin to the mice for long duration by iPRECIO pump

(**a**) Schedule of blood sampling from mouse tail for luciferin assay. Luciferin administration (40 mg ml^{-1}) was started in the morning on day 0. A iPRECIO pump was connected to the freely moving mice system and the tube was guided into intraperitoneal cavity (Supplementary Fig. 1g). **(b)** Representative chromatogram of luciferin in the plasma from mouse tail, obtained with a chiral column. (**c**) Luciferin concentration (mean ± s.d.) on day 2-3 supplying at various pump supplying speeds. Collected plasma luciferin in the blood from the mouse tail was measured by HPLC. In all cases, luciferin concentrations were stable. (**d**) Luciferin concentration (mean ± s.d.) on day 6-7. Luciferin concentrations on days 6-7 were the same as days 2-3.

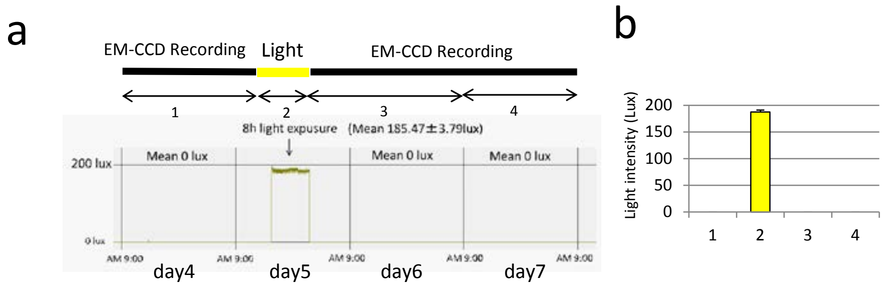

Supplementary Figure 10a-b

Supplementary Figure 10

Photic resetting of *Per1* expression by an 8 hour light exposure

(**a**) Experimental protocol of 8 hour light pulse. Mice were given an 8 hour light pulse from CT 12 on day 5. Images were captured for 10 min, every 0.5 sec within each 0.5 hour interval. Recording of *Per1* gene expression was stopped during light pulse exposure. Light intensity (lux) in the recording cage is shown in (**b**). Mean light intensity during 8 hour light pulse exposure. The abscissa indicates the duration of time in (a).

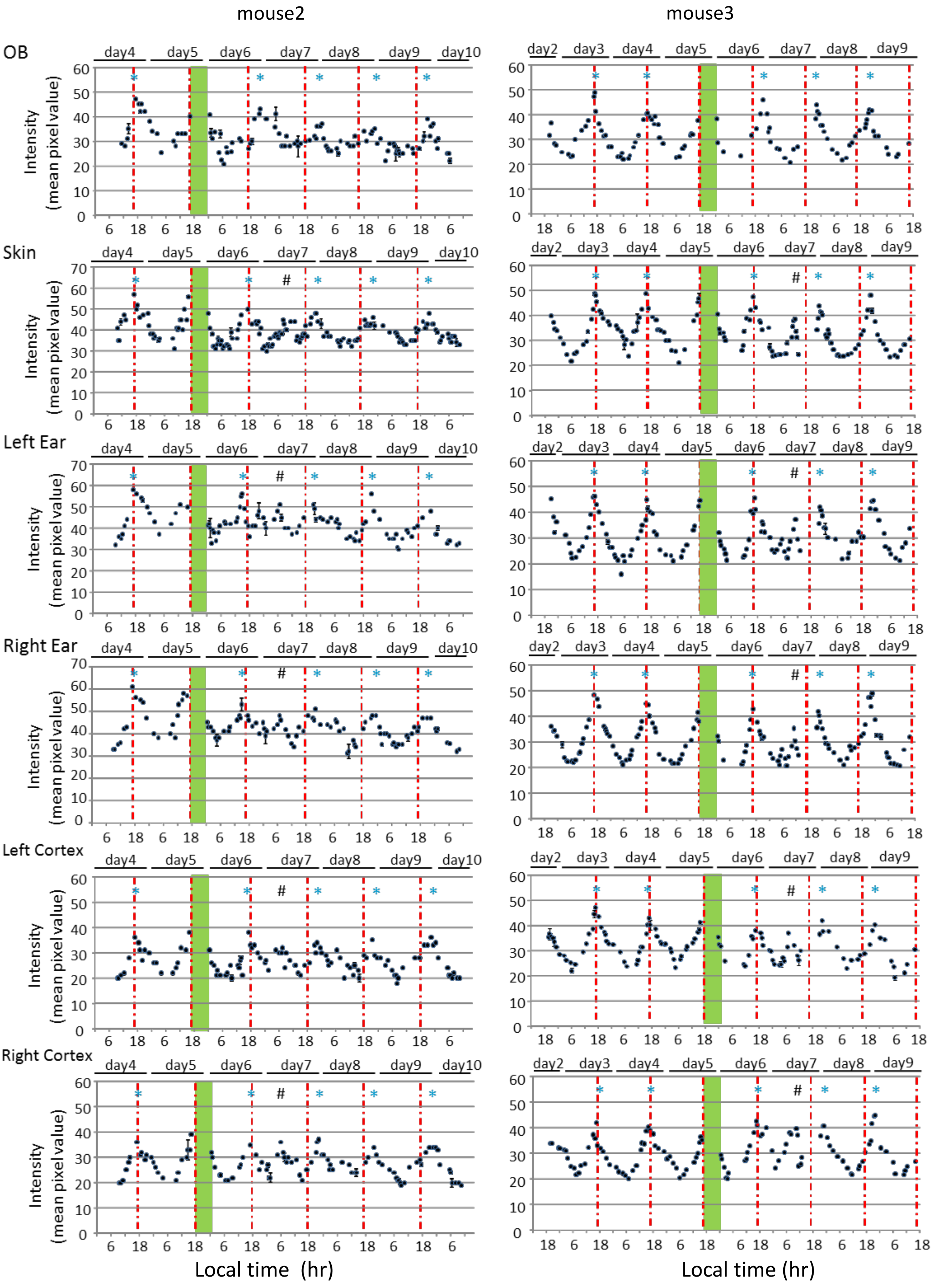

Supplementary Figure 11

Supplementary Figure 11

Photic resetting of *Per1* expression in 6 different areas

In vivo imaging of *Per1* expression in 6 areas of *Per1-luc* mouse2 and *Per1-luc* mouse3.

Supplementary Table1. Cosinor analysis of period, $F_{(2,14)}$ -value, p -value, best fitting (%) in *Per1* expression of *Per1-luc* mice.

	Period,	$F_{(2,14)}$ -value,	p -value,	Best fitting (%)
OB	24.0 h	67.9	4e-6	89.0
Skin	24.0 h	18.1	2.8e -4	67.0
left ear	24.0 h	67.7	4e-6	88.7
right ear	24.0 h	19.6	2.1e-4	68.6
left cortex	24.0 h	68.1	4e -6	89.0
right cortex	24.0 h	61.6	6e -6	87.9

Supplementary Table2. Cosinor analysis of mesor, amplitude, acrophase in *Per1* expression *Per1-luc* mice.

	Mesor,	amplitude,	acrophase
OB	50.0	11.1	18 h 29 min
Skin	65.2	18.0	18 h 35 min
left ear	65.2	18.0	18 h 35 min
right ear	53.2	8.6	17 h 40 min
left cortex	50.0	12.7	17 h 56 min
right cortex	50.3	12.9	17 h 36 min

Supplementary Table3. Cosinor analysis of period, $F_{(2,14)}$ -value, p -value, best fitting (%) in *Per1* expression of *Cry1*$^{-/-}$/*Cry2*$^{-/-}$-*Per1-luc* mice.

	$F_{(2,14)}$ -value,	p -value,	Best fitting (%)
OB	0.2	0.9	0.0
Skin	1.4	0.3	0.0
left ear	2.2	0.1	5.5
right ear	0.0	1.0	0.0
left cortex	0.8	0.5	0.0
right cortex	0.1	0.9	0.0
