# Supplementary figures and images for: In vivo imaging of clock gene expression in multiple tissues of freely moving mice
Source: Nat Commun. 2016 Jun 10;7:11705. doi: 10.1038/ncomms11705 (PMC5446038; doi:10.1038/ncomms11705)

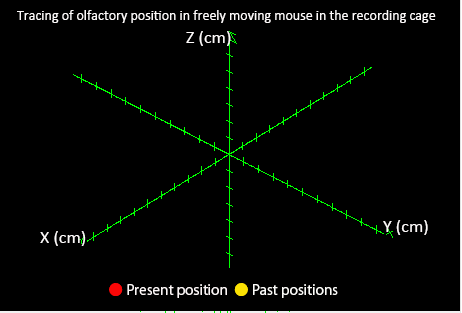

Supplement: Supplementary Movie 1 — Tracking of a scintillator placed on the olfactory bulb [file ncomms11705-s2.gif]
